# MetaCGRP is a high-precision meta-model for large-scale identification of CGRP inhibitors using multi-view information

**DOI:** 10.1038/s41598-024-75487-x

**Published:** 2024-10-21

**Authors:** Nalini Schaduangrat, Phisit Khemawoot, Apisada Jiso, Phasit Charoenkwan, Watshara Shoombuatong

**Affiliations:** 1 Center for Research Innovation and Biomedical Informatics, Faculty of Medical Technology, Mahidol University, Bangkok, 10700 Thailand; 2 Chakri Naruebodindra Medical Institute, Faculty of Medicine Ramathibodi Hospital, Mahidol University, Samut Prakan, 10540 Thailand; 3 Modern Management and Information Technology, College of Arts, Media and Technology, Chiang Mai University, Chiang Mai, 50200 Thailand

**Keywords:** Calcitonin gene-related peptide, QSAR, Cheminformatics, Machine learning, Feature selection, Meta-model, Computational biology and bioinformatics, Data mining, Machine learning

## Abstract

Migraine is considered one of the debilitating primary headache conditions with an estimated worldwide occurrence of approximately 14–15%, contributing highly to factors responsible for global disability. Calcitonin gene-related peptide (CGRP) is a neuropeptide that plays a crucial role in the pathophysiology of migraines and thus, its inhibition can help relieve migraine symptoms. However, conventional process of CGRP drug development has been laborious and time-consuming with incurred costs exceeding one billion dollars. On the other hand, machine learning (ML)-based approaches that are capable of accurately identifying CGRP inhibitors could greatly facilitate in expediting the discovery of novel CGRP drugs. Therefore, this study proposes a novel and high-accuracy meta-model, namely MetaCGRP, that can precisely identify CGRP inhibitors. To the best of our knowledge, MetaCGRP is the first SMILES-based approach that has been developed to identify CGRP inhibitors without the use of 3D structural information. In brief, we initially employed different molecular representation methods coupled with popular ML algorithms to construct a pool of baseline models. Then, all baseline models were optimized and used to generate multi-view features. Finally, we employed the feature selection method to optimize the multi-view features and determine the best feature subset to enable the construction of the meta-model. Both cross-validation and independent tests indicated that MetaCGRP clearly outperforms several conventional ML classifiers, with accuracies of 0.898 and 0.799 on the training and independent test datasets, respectively. In addition, MetaCGRP in conjunction with molecular docking was utilized to identify five potential natural product candidates from Thai herbal pharmacopoeia and analyze their binding affinity and interactions to CGRP. To facilitate community-wide efforts in expediting the discovery of novel CGRP inhibitors, a user-friendly web server for MetaCGRP is freely available at https://pmlabqsar.pythonanywhere.com/MetaCGRP.

**Supplementary Information:**

The online version contains supplementary material available at 10.1038/s41598-024-75487-x.

## Introduction

Migraine is considered one of the debilitating primary headache conditions according to the 3rd edition of the International Classification of Headache Disorders (ICHD-3)^1^. In the Global Burden of Disease Study 2021 (GBD2021), headache disorders were positioned as the 15th leading cause of disability-adjusted life years (DALYs) worldwide, with migraine accounting for greater than 80% of these headache disorders^2–5^. The estimated worldwide occurrence of migraine is approximately 14–15%, contributing to 4.9% of the factors responsible for global disability^6^. Migraine tends to be more prevalent in women due to hormonal influences and is characterized by a throbbing headache, typically ranging in severity from moderate to severe^7^. Of note, current preventive medications, such as beta-blockers, antiepileptic drugs, angiotensin receptor blockers, and antidepressants, lack specificity for migraine treatment. Additionally, their usage is often constrained due to the presence of side effects and contraindications^8^.

Over-the-counter analgesics including non-steroidal anti-inflammatory drugs (NSAIDs) are frequently use in acute migraine treatment. Triptans, selective 5-hydroxytryptamine1B/1D (5-HT1B/1D) receptor agonist, are the second line therapy for migraine treatment. Due to 5-HT1B receptor–mediated vasoconstriction, triptans are contraindicated in people with cardiovascular risks. In the case of treatment failure, the third line therapy drug, 5-hydroxytryptamine type 1 F (5-HT1F) receptor agonists or ditans and calcitonin gene-related peptide (CGRP) antagonists are used^9^.

CGRP is a neuropeptide consisting of 37 amino acids and serves as a potent vasodilator and neurotransmitter within both the peripheral and central nervous systems. CGRP is released from the trigemino-vascular nociceptive system and plays a crucial role in the pathophysiology of migraines as evidenced by elevated serum levels of CGRP during a migraine attack^10,11^. The CGRP receptor is a heterodimeric G-protein coupled receptor, comprising receptor activity-modifying protein type 1 (RAMP1) and calcitonin-like receptor (CLR). The N-terminal extracellular domain (ECD) of this receptor is a pivotal component in the binding of ligands^12^. A comprehensive review discussing the role of CGRP in migraines has been published^13^. For the treatment of preventive and acute migraine conditions, the FDA has approved drugs targeting CGRP. These include CGRP receptor antagonists, known as the gepants group (i.e., ubrogepant, rimegepant, atogepant, and zavegepant), as well as CGRP monoclonal antibodies (mAbs) like erenumab-aooe, fremanezumab, galcanezumab-gnlm, and eptinezumab-jjmr^14–16^. However, side effects such as upper respiratory tract infections, urinary tract infections, joint stiffness, liver toxicity to name a few, from these drugs are still observed^17^. Hence, the development of CGRP inhibitors with fewer side effects are still a need of the hour. Innovative drug developments aimed at targeting the CGRP receptor continue to emerge. The market value of drugs targeting CGRP is projected to exceed $6.5 billion by 2027^18^. However, conventional process of drug development has been complicated, time-consuming (spanning 12–15 years), and incurring costs exceeding one billion USD^19^. A valuable tool in drug design, known as computer-aided drug design (CADD), encompasses both structure-based drug design and ligand-based drug design. It employs various methodologies, including quantitative structure-activity relationship (QSAR), machine learning (ML), molecular dynamics (MD), molecular docking, combined quantum mechanics/molecular mechanics (QM/MM) simulations, and deep learning (DL)^20^.

Natural products hold great potential as a source for the development of new drugs. The intricate and diverse structures found in natural products have evolved over time in organisms to serve as defense mechanisms and facilitate interactions with other organisms^21^. The fusion of natural product research and ML has seen significant advancements. ML effectively leverages chemical information from natural products for tasks such as identifying active compounds, conducting spectral and chromatogram analyses, and even automatically designing compounds resembling natural products^22^. ML algorithms have also been instrumental in exploring the bioactivity of natural products, including their effectiveness in areas such as antimicrobial, anti-malarial, anti-cancer, and anti-inflammatory activities^23^. More recently, ML-based approaches have been applied in Traditional Chinese medicine (TCM) research for purposes ranging from disease diagnosis and treatment to evaluating treatment effects and predicting biomarkers within TCM^24^. In clinical trials, compounds like ginkgolide b from Ginkgo biloba, root extracts from Petasites hybridus (butterbur), and Tanacetum parthenium (feverfew) have demonstrated anti-migraine properties^25^. However, there have been no reported instances of natural products specifically targeting the CGRP receptor. The existing literature only includes ML-based predictions of migraine patient responses over a 6–12-month period^26^.

**Fig. 1:**
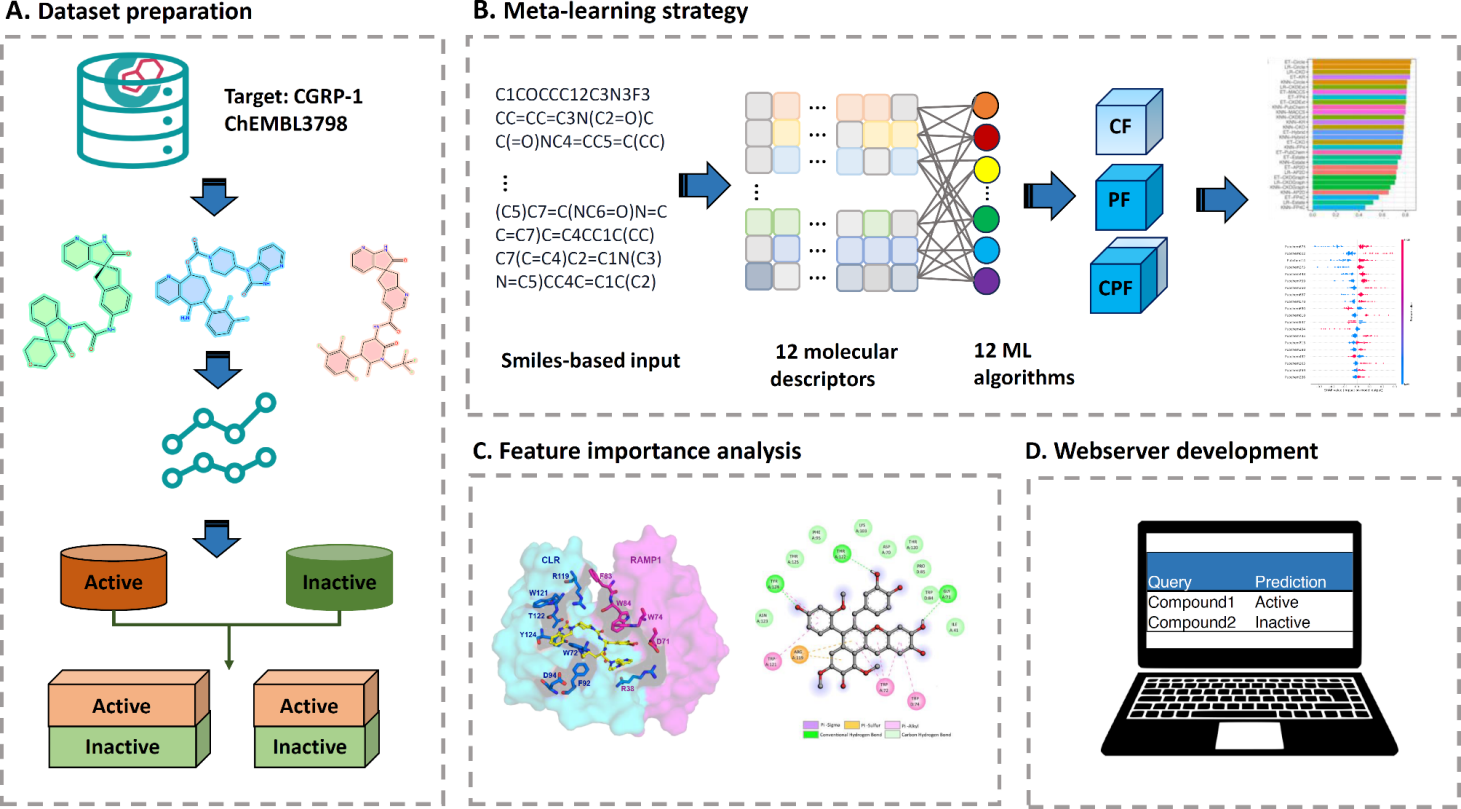
An overview of MetaCGRP for identifying CGRP inhibitors using only the SMILES notation. The construction of MetaCGRP involves four primary steps, which include (i) preparing the training and independent test datasets, (ii) building a pool of baseline models, (iii) generating multi-view feature representations, and optimizing the meta-learning model, and (iv) analyzing the performance and feature importance.

Taking all this into account, herein, we propose a high-accuracy computation approach, named MetaCGRP, by employing the meta-learning strategy. To the best of our knowledge, MetaCGRP is the first computational approach that has been developed to identify CGRP inhibitors using only the SMILES notation. The development and evaluation processes of MetaCGRP are illustrated in Fig. 1. As can be seen from Fig. 1, MetaCGRP employed 12 different molecular representation methods to encode CGRP inhibitors into fix-length feature vectors. Then, these feature vectors were used to develop a pool of baseline models. Second, we utilized such baseline models to generate multi-view features providing two crucial aspects, involving category information and probability information. Finally, to enhance the performance of the proposed model, the feature selection method was applied to optimize the multi-view features and the best feature set was then used for to construct the final meta-model. Extensive experiments on the independent test dataset show that MetaCGRP attained superior predictive performance than several conventional ML classifiers, with an accuracy (ACC) of 0.897, area under the receiver-operating curves (AUC) of 0.938, and Matthew’s coefficient correlation (MCC) of 0.799. In addition, SHapley Additive exPlanations (SHAP) was employed to disclose the feature importance information. An analysis of the top 20 important substructure features for CGRP identification revealed that features consisting of nitrogen and halogen substituents as well as those derived from natural products were the most significant. Finally, we utilized MetaCGRP coupled with molecular docking to identify five potential natural product candidates from Thai herbal pharmacopoeia and then analyze their binding affinity and interactions to CGRP.

## Materials and methods

### Dataset construction

Herein, the ChEMBL database was used for collecting compounds pertaining to our target (i.e., CGRP; ID: ChEMBL3798)^27^. Initially, we retrieved a total of 1509 compounds with activity towards CGRP-1, and then subjected them to data preprocessing using our in-house scripts using the R programming environment^28^. During this process, compounds with ‘=’ symbols in their bioactivity unit column were retained, while those containing symbols such as ‘<‘, ‘>’, or ‘/‘ were excluded. Additionally, missing and duplicate data points were eliminated. Subsequently, we refined the dataset by selecting compounds with a bioactivity unit of IC_50_ and a standard deviation of 2. This resulted in a final dataset comprising 389 compounds. To enhance data clarity and facilitate comparisons of drug potency at equivalent molar concentrations^29^, we converted these compounds into their pIC_50_ values (negative logarithm base 10 of IC_50_ in Molar concentration). Specifically, compounds with pIC_50_ values greater than 6 were categorized as active, while those with pIC_50_ values less than 6 were classified as inactive. Compounds with pIC_50_ values falling in between were considered as intermediate and were not used in this study so as to mitigate noise introduced into the classification models with borderline values as summarized in^30,31^. Consequently, the final dataset consisted of 181 active compounds and 135 inactive compounds, respectively. Finally, the 181 active and 135 inactive compounds were randomly divided to create training (144 active and 108 inactive compounds) and independent test (37 active and 27 inactive compounds) datasets in an 8:2 ratio.

### Chemical space analysis

Studying the chemical landscape proves to be a valuable method to explore, comprehend, and enhance the vast range of potential compounds, all with the goal of discovering prospective drug candidates. As stated earlier, we classified all molecules into two groups, active and inactive, depending on their pIC_50_ values. In this context, we computed, visualized, and examined six physicochemical characteristics linked to Lipinski’s Rule of Five (Ro5)^32^ and Veber’s^33^ rule for both classes of bioactivity. These properties encompass the Ghose-Crippen-Viswanadhan octanol-water partition coefficient (ALogP), molecular weight (MW), the count of hydrogen-bond donors (HBDon), topological polar surface area (TPSA), number of rotatable bonds (nRotB) and the count of hydrogen-bond acceptors (HBAc). To create graphical representations of the mentioned descriptors, we employed in-house scripts utilizing the ‘ggplot2’ package within the R programming environment (version 4.2.2)^28^. Furthermore, we conducted statistical analyses, which included calculating maximum, minimum, mean, and median values, and the statistical significance was evaluated using p-values obtained from the Mann-Whitney U test.

Chemical diversity refers to the variety of chemical structures present within a dataset. In drug discovery, chemical diversity is a crucial factor because it affects the generalizability, robustness, and success of computational models. Here, chemical diversity was assessed by computing the ECFP4 fingerprints^34^ of all compounds and calculating the Tanimoto similarity, which is a measure commonly used in cheminformatics to compare the similarity between two chemical structures of the dataset, as noted in^35^. A clustered heatmap of the similarity matrix was created using ‘pheatmap’ package in the R programming environment^28^.

### Molecular representation method

Molecular descriptors, namely molecular representations and fingerprints, can be defined as both quantitative and qualitative representations of molecules of interest, encompassing their structural makeup, connections, and physicochemical properties^36^. These descriptors play a crucial role in quantitative structure-activity relationship (QSAR) investigations^29^. In our research, we conducted data pre-processing steps, such as eliminating salts, eliminating duplicate data, and standardizing tautomers, utilizing the built-in functionality of the PADEL-descriptor software^37^. Following this pre-processing phase, we used the SMILES notation of the compounds under examination as input for generating molecular fingerprints. We employed a total of 12 distinct fingerprint types in this study, which include FP4, PubChem, Estate, CDKExt, CDKGraph, AP2D, Circle, CDK, Hybrid, KR, FP4C, and MACCS^38–45^. For a comprehensive understanding of each fingerprint, please refer to Table 1. All calculations related to molecular descriptors were executed within the Python programming environment^46^.

**Table 1:** Summary of twelve molecular representation methods used in this study.

Fingerprint	Abbreviation	#Feature	Description	Ref
2D atom pair	AP2D	780	Presence of atom pairs at various topological distances	^38^
CDK	CDK	1,024	Fingerprint of length 1,024 and search depth of 8	^41^
CDK extended	CDKExt	1,024	Extends the fingerprint with additional bits describing ring features	^41^
CDK graph only	CDKGraph	1,024	A special version that considers only the connectivity and not bond order	^41^
Circle	Circle	1,024	Circular fingerprint	^40^
EState	EState	79	Electrotopological state atom types	^42^
Hybrid	Hybrid	1,024	CDK hybridization fingerprint	^40^
Klekota–Roth	KR	4,860	Presence of chemical substructures	^39^
MACCS	MACCS	166	Binary representation of chemical features defined by MACCS keys	^44^
Pubchem	PubChem	881	Binary representation of substructures defined by PubChem	^43^
Substructure	FP4	307	Presence of SMARTS patterns for functional groups	^45^
Substructure count	FP4C	307	Count of SMARTS patterns for functional groups	^45^

### Meta-learning strategy

Numerous studies have demonstrated that the meta-learning approach is able to provide improved performance compared to conventional ML methods^47–52^. In this strategy, it involves three primary steps: (i) building a pool of baseline models, (ii) generating multi-view feature representations, and (iii) optimizing the meta-learning model (Fig. 1).

#### Building a pool of baseline models

In the first step, we employed 12 conventional molecular representation methods (i.e., FP4, Pubchem, Estate, CDKExt, CDKGraph, AP2D, Circle, CDK, Hybrid, KR, FP4C, and MACCS) to encode positive and negative samples on the CGRP-TRN dataset. We subsequently applied each molecular descriptor to train 12 individual classifiers by using 12 popular ML algorithms (i.e., SVM, KNN, LR, DT, ET, MLP, NB, PLS, XGB, RF, LGBM, and ADA), where each classifier developed in this step was called a baseline model. The details of all the molecular descriptors and ML methods are provided in Tables 1 and 2. To date, these ML methods and conventional molecular descriptors have been successfully applied in drug discovery and development^52–55^. As a result, a total of 144 baseline models were trained and constructed using the scikit-learn package^47–49,51^. To obtain robust baseline models, their parameters were optimized and selected using a grid search and the 10-fold cross-validation strategy.

**Table 2:** Information of parameter settings for 12 ML methods used in this study.

Method	Parameter	Search space
ADA	n_estimators	[20, 50, 100, 200, 500]
DT	max_depth	2–20 with an interval of 1.
ET	n_estimators	[20, 50, 100, 200, 500]
KNN	number of neighbours	1–150 with an interval of 1
LGBM	n_estimators	[20, 50, 100, 200, 500]
LR	C	np.logspace(-3, 3, num = 100)
MLP	hidden_layer_sizes	[20, 50, 100, 200, 500]
NB	var_smoothing	np.logspace(0,-9, num = 100)
PLS	#Components	10–1000 with an interval of 10
RF	n_estimators	[20, 50, 100, 200, 500]
SVM	Cost	[2^− 4^–2^4^] in log_2_ steps
XGB	n_estimators	[20, 50, 100, 200, 500]

Columns 2 and 3 represents the parameter name used in the Scikit-learn library and the range of parameter used to develop the model, respectively.

#### Generating multi-view feature representations

After obtaining the well-trained 144 baseline models, we utilized them to generate multi-view feature representations. Herein, each baseline model will provide the prediction outputs proving category information and probability information. The corresponding output were considered as class and probabilistic features. For a given compound, its class and probabilistic feature vectors were represented with a 144-D (CF) and 144-D (PF) feature vectors, respectively. To maximize the utilization of both information, we combined CF and PF to enable the construction of CPF, which was represented with a 288-D feature vector.

#### Optimizing meta-models

In the last step, we investigated the performance of three multi-view feature representations in CGRP inhibitor identification by developing three individual XGB-based meta-model (mXGB) models coupled with CF, PF, and CPF. Here, our powerful genetic algorithm (GA-SAR) was used to optimize all the CF, PF, and CPF in order to improve their discriminative ability and predictive performance of the three mXGB models. The GA-SAR’s chromosome involves binary for feature optimization and parametric genes for mXGB parameter optimization. As a results, the GA-SAR’s chromosome used herein comprises 144 genes and 3-bit gene for encoding the n_estimators parameter (Table 2). Specifically, the parameters and their values for the GA-SAR used herein contain *r*_*begin*_ = 5, *m*_*stop*_ = 50, *P*_*m*_ = 0.05, and *Pop* = 20^48,49,51^. Detailed information about the GA-SAR algorithm is summarized in our previous articles^48,51,56^. Finally, the feature representation providing the highest cross-validation MCC was selected for the construction of the final meta-model^57–61^. In addition, we employed a total of six performance metrics, including ACC, MCC, AUC, F1, sensitivity (Sn), and specificity (Sp)^62–68^ to evaluate and compare the performance evaluation results of MetaCGRP and its baseline models. More information regarding these six performance metrics is provided in **Supplementary information.**

### Molecular docking

The 3D structure of the CGRP receptor ectodomain complex (PDB ID: 3N7S)^69^, along with its interaction with olcegepant (3N6), was sourced from the RCSB Protein Data Bank (PDB; https://www.rcsb.org/). The virtual screening dataset was obtained from the Thai herbal pharmacopoeia (https://bdn.go.th/thp/home ; accessed on 18 June 2023) and contained a total of 353 natural compounds. The SMILES of each compound was manually curated from Pubchem (https://pubchem.ncbi.nlm.nih.gov/) and used as input for our meta model. The top 15 compounds with the highest probability were then subjected to molecular docking. In preparation for the protein analysis, we eliminated ligand and water molecules and introduced polar hydrogen atoms and charges. Subsequently, the ligand and protein files were transformed into the PDBQT format using MGLTools^46^. Docking experiments involving the selected structures were carried out using Autodock Vina^70^. We assessed the reliability of the docking criteria by re-docking the native ligand (3N6) and comparing the docking position to its corresponding crystallographic inhibitor, resulting in a root-mean-square deviation (RMSD) of 1.34 Å which is suitable for further investigations. For the docking simulations, we established the center site of CLR/RAMP1 with coordinates x = 22.696, y = 21.496, and z = 72.184 (in Å) within a grid box of dimensions X: 62, Y: 56, and Z: 60 (in Å). The exhaustiveness parameter was set to 32, and an energy range of 4 was specified. Subsequently, we chose the best binding affinities (in kcal/mol) of the compounds based on their more negative values from the docking results. Finally, we visualized the interactions and binding poses of the top 5 compounds using PyMOL (Schrodinger, Inc.).

## Results and discussion

### Chemical space analysis of CGRP inhibitors

The chemical space analysis conducted herein focused on understanding the unique characteristics between the bioactivity classes (i.e., active and inactive) of CGRP inhibitors. Over time, guidelines for drug-like molecules have been formulated by the pharmaceutical industry and medicinal chemists, anticipating their permeability across biological membranes^32,33^. This exploration employed Veber’s and Lipinski’s Ro5, including AlogP ≤ 5, MW ≤ 500, HBDon ≤ 5, HBAc ≤ 10, TPSA ≤ 140 Å^2^, and nRotB ≤ 10. Although most small molecules that are orally available tend to follow the Ro5 criteria, many exceed these boundaries^71^. This observation suggests that these criteria are based on the understanding that effective medications often comprise larger molecules with a certain degree of lipophilicity^72^.

MW represents the compound’s mass and is commonly used for mathematical calculations and interpretations, whereas AlogP serves as an established parameter that gauges a molecule’s hydrophobicity, or lipophilicity, offering insights into its capability to enter and traverse cell membranes. The counts of HBDon and HBAc are employed to evaluate a molecule’s ability to form hydrogen bonds (H-bond). Additionally, TPSA is linked to the pattern of hydrogen bonding of a molecule under investigation in an aqueous environment, while nRotB is associated with molecules with flexibility that establish intrachain hydrogen bond interactions, thereby enhancing permeation into cell membranes^73,74^. Supplementary Figure S1 illustrates the chemical space using a combination of box and violin plots for all the descriptors mentioned above. Supplementary Figure S1A shows a concentrated distribution of active compounds, with a mean molecular weight of 600 Da and an approximate range of 550 to 650 Da, while the inactive compounds are clustered between 450 and 620 Da, with a mean of 550 Da. The AlogP of active compounds show a range of 2 to 6 with a mean of 4 while the inactive compounds fall in the range of 1 to 7 with a mean at 3 (Supplementary Figure S1B). Additionally, our statistical analysis, using the Mann–Whitney U test, found a significant difference between the active and inactive compounds (p-value < 0.001) for the AlogP descriptor, but no statistical significance for MW between the groups.

The visualization further emphasizes that most compounds possess HBDon values under 5 and HBAc values below 10. A significant statistical difference (p-value < 0.001) between active and inactive compounds was noted only for the HBAc property, indicating a higher number of H-bond acceptors in the active compounds (Supplementary Figure S1C-S1D). Similarly, the distribution of active and inactive compounds in terms of TPSA shows a trend with a mean value at 120 Å^2^ (Supplementary Figure S1E). Although the nRotB of most compounds in both classes were within the desired range (i.e., less than 10), their distribution was observed to be statistically significant with a p-value < 0.001 (Supplementary Figure S1F).

However, it’s important to note that for most compounds in the dataset, their MW surpasses the threshold defined by Ro5. This deviation can be attributed to the nature of the CGRP receptor, which consists of two distinct domains, CLR and RAMP1. To effectively inhibit or bind to the CGRP receptor, a molecule needs to interact with both of these components, necessitating a larger molecular size^69^. An illustration of this is the potent inhibitor olcegepant, which has a MW of 869.645 Da (DrugBank ID: DB04869). Additionally, FDA approved small-molecule drugs for CGRP such as rimegepant, ubrogepant, atogepant, and zavegepant have MWs of 534.6, 549.5, 603.5, and 638.8 Da, respectively, all of which exceed the Ro5 rule of 500 Da. This might be a result of larger molecules containing extra hydrophobic regions or functional groups, making them more likely to partition into organic phases such as octanol. Moreover, larger molecules with higher MW have a greater potential for establishing numerous interactions with the target molecule^75^.

Furthermore, to assess the chemical diversity of the dataset, Tanimoto similarity was calculated and represented as a heatmap, illustrated in Supplementary Figure S2. The color of each cell in the heatmap corresponds to the Tanimoto similarity score between the molecules represented by the respective row and column. Cells with colors representing higher values (red) indicate high similarity (values closer to 1), while cells with lighter or less intense colors (blue) indicate low similarity (values closer to 0). In addition, the diagonal of the heatmap (from the top left to the bottom right) signifies the comparison of each molecule with itself, and should have a Tanimoto similarity score of 1 (or 100%), indicating perfect similarity. As can be seen from Supplementary Figure S2, the diagonal line is uniformly dark red, as each molecule is identical to itself. There are also several off-diagonal blocks that range from dark red to red, orange, and yellow, indicating clusters of molecules that are highly and moderately similar to each other. Finally, outside these blocks, the heatmap is mostly blue, suggesting that these clusters are quite different from the rest of the dataset. This interpretation suggests that while there are some groups of similar molecules, the dataset overall contains a good amount of diversity, which is generally favorable for creating robust and generalizable models.

### Comparison of different ML methods and molecular representation methods

In this section, we evaluated and analyzed the impact of several baseline models trained on the 12 powerful ML methods and 12 conventional molecular descriptors in the identification of CGRP inhibitors by performing both 10-fold cross-validation and independent tests. The performance of these prediction models is recorded in Supplementary Tables S1-S2 while Supplementary Tables S3-S6 provide the average performance of each ML method and molecular descriptors. As seen in Supplementary Table S3, the highest average cross-validation MCC of 0.765, 0.764, 0.758, 0.752, and 0.752 are obtained from LR, XGB, ET, RF, and SVM, respectively. Based on the performance of the 12 molecular descriptors, the highest average cross-validation MCC of 0.722, 0.735, 0.736, 0.752, and 0.777 are obtained from FP4, CKDExt, CKD, Circle, and Hybrid, respectively (Supplementary Table S5). In addition, Supplementary Figure S3 highlights the chemical space in terms of applicability domain of the 12 molecular descriptors as visualized using the t-distributed Stochastic Neighbor Embedding (t-SNE) approach. The compounds in the independent dataset are observed to form clusters in the same areas as compounds from the training dataset, indicating their similarity and reliability for prediction purposes. This suggests that these ML methods and molecular descriptors are beneficial in CGRP inhibitor identification.

**Fig. 2:**
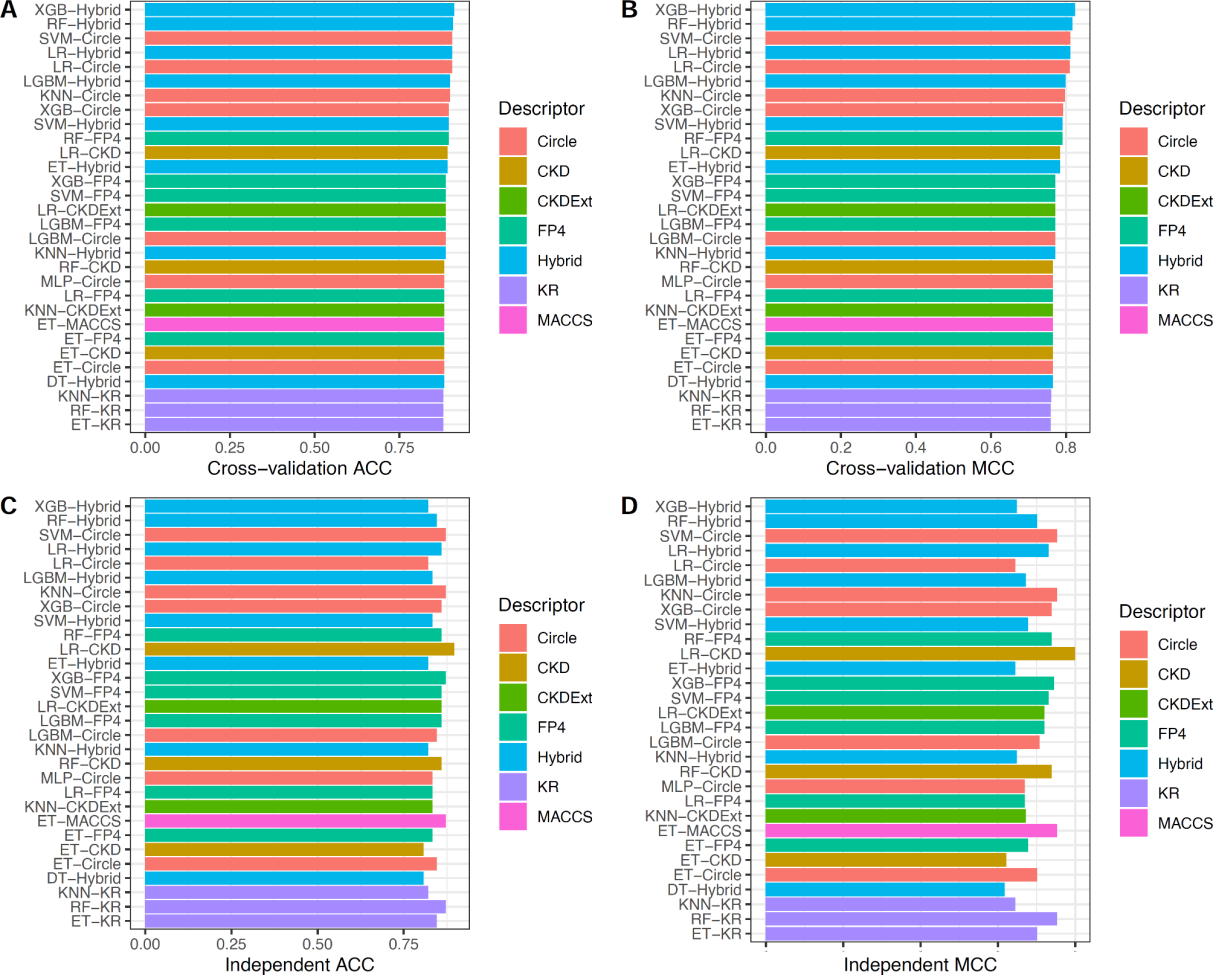
Performance comparison of top 20 baseline models as evaluated using training (**A-B**) and independent (**C-D**) datasets.

Furthermore, we compared the performance of 144 baseline models and determined the best-performing one as judged by cross-validation MCC. From Fig. 2 and Supplementary Tables S1-S2, several observations can be summarized as follows: (i) The top-five powerful baseline models consist of XGB-Hybrid, RF-Hybrid, SVM-Circle, LR-Hybrid, and LR-Circle with respective cross-validation MCC of 0.824, 0.817, 0.811, 0.811, and 0.810; (ii) Among the top-five powerful baseline models, all of them are developed by using Hybrid and Circle. These observations again confirm the discriminative ability of these two molecular descriptors; and (iii) We noticed that XGB-Hybrid outperforms other prediction models in terms of cross-validation MCC. On the other hand, MLP-Pubhem (MCC of 0.804) outperforms XGB-CDK (MCC of 0.649) as judged by the MCC of the CGRP-IND dataset. These observations indicate that the performance of single-based feature descriptors is not robust as indicated by the CGRP-IND dataset. Thus, it is desirable to develop a more robust model by using the meta-learning strategy.

### Comparison of different multi-view feature representations

In this study, we employed three multi-view feature representations, including CF, PF, and CPF, to address the limitation of the single feature descriptor. Furthermore, we applied the GA-SAR method to each feature representation for improving its discriminative ability. Herein, the GA-SAR method identified 10, 19, and 8 informative features for constructing the best feature sets for CF, PF, and CPF, respectively. For convenience of discussion, the best feature sets for CF, PF, and CPF are referred as CF_FS, PF_FS, and CPF_FS, respectively. To assess the feature ability of six feature representations in the identification of CGRP inhibitors, both 10-fold cross-validation and independent tests were used on the CGRP-TRN and CGRP-IND datasets, respectively. The prediction performance of the six new feature representations is recorded in Table 3. As seen in Table 3, all the six metrics of CF_FS, PF_FS, and CPF_FS are higher than that of their original feature vectors on the CGRP-TRN dataset. Furthermore, the MCC values of CF_FS, PF_FS, and CPF_FS were 5.65, 9.44, and 8.12, % higher than their original feature vectors, respectively. Among the three optimal feature representations, the performance of PF_FS was slightly higher than CF_FS and CPF_FS over the 10-fold cross-validation test. In case of the independent test, PF_FS secures the best performance in terms of all the six measures. To be specific, the ACC, SP, MCC, AUC, and F1 of PF_FS were 2.56–3.85, 2.26–5.13, 2.56, 5.16–7.52, 0.79–1.26, and 2.70–4.26% higher than the compared feature representations. Overall, PF_FS showed a stable performance on both the CGRP-TRN and CGRP-IND datasets. Therefore, the PF_FS was selected as input feature vector to develop our proposed meta-model (named MetaCGRP).

**Table 3:** Cross-validation and independent test results of different feature representations.

Evaluation strategy	Feature	ACC	SN	SP	MCC	AUC	F1
Cross-validation	CF	0.918	0.914	0.922	0.837	0.948	0.917
	PF	0.902	0.901	0.903	0.804	0.941	0.901
	CPF	0.902	0.901	0.903	0.804	0.941	0.901
	CF_FS	0.945	0.948	0.942	0.893	0.961	0.945
	PF_FS	0.948	0.967	0.928	0.898	0.958	0.949
	CPF_FS	0.941	0.928	0.955	0.885	0.959	0.939
Independent test	CF	0.872	0.821	0.923	0.748	0.897	0.865
	PF	0.846	0.795	0.897	0.696	0.934	0.838
	CPF	0.846	0.795	0.897	0.696	0.934	0.838
	CF_FS	0.859	0.795	0.923	0.724	0.930	0.849
	PF_FS	0.897	0.846	0.949	0.799	0.938	0.892
	CPF_FS	0.872	0.821	0.923	0.748	0.921	0.865

### Meta-learning strategy is capable of contributing to performance improvement

**Fig. 3:**
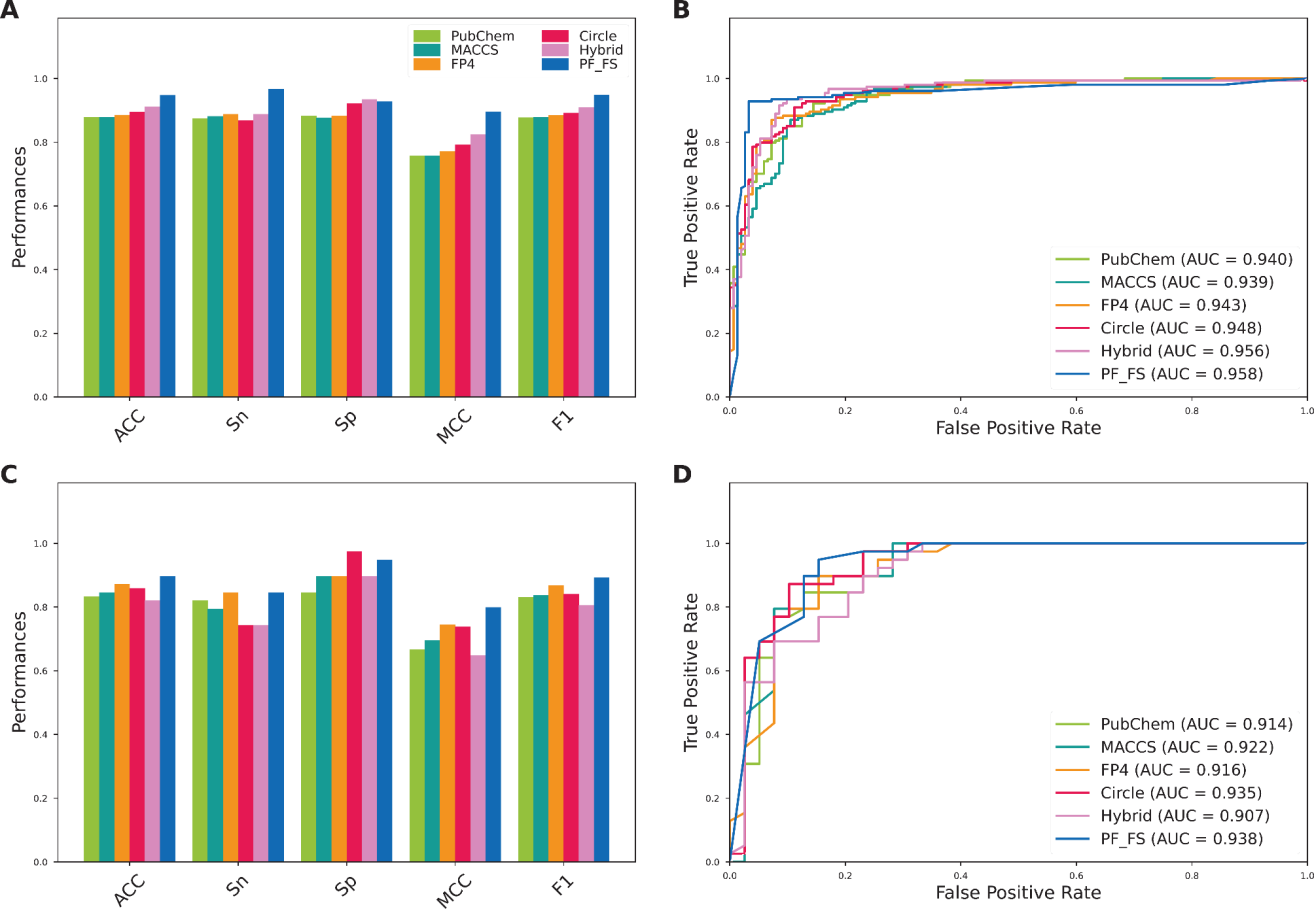
Performance comparison of our generated feature PF_FS and top-five molecular descriptors on the training (**A–B**) and independent test (**C–D**) datasets.

To reveal the advantages of the meta-learning strategy, we investigate and evaluate the feature ability of our new feature representations (PF_FS) and the effectiveness of the proposed model (MetaCGRP) by conducting two individual comparative experiments. In the first comparative experiment, we compared the performance of the PF_FS with 12 conventional molecular descriptors. For a fair comparison, we trained and evaluated XGB classifiers with these molecular descriptors in terms of both 10-fold cross-validation and independent tests. The performance evaluation results are provided in Table 4. Additionally, Fig. 3 highlights the performance comparison of PF_FS with the top-five feature descriptors having the highest cross-validation MCC (i.e., Pubchem, MACCS, FP4, Circle, and Hybrid). It can clearly be seen that PF_FS attained better performance than the top-five feature descriptors on both the CGRP-TRN and CGRP-IND datasets.

**Table 4:** Cross-validation and independent test results of different feature descriptors.

Evaluation strategy	Feature	ACC	SN	SP	MCC	AUC	F1
Cross-validation	FP4C	0.830	0.829	0.831	0.660	0.911	0.829
	CKDGraph	0.840	0.836	0.844	0.680	0.923	0.838
	AP2D	0.846	0.855	0.838	0.693	0.922	0.847
	Estate	0.850	0.868	0.831	0.700	0.906	0.852
	KR	0.856	0.882	0.831	0.713	0.938	0.859
	CKD	0.866	0.862	0.870	0.732	0.943	0.865
	CKDExt	0.869	0.862	0.877	0.739	0.950	0.868
	PubChem	0.879	0.875	0.883	0.758	0.946	0.878
	MACCS	0.879	0.882	0.877	0.758	0.938	0.879
	FP4	0.886	0.888	0.883	0.771	0.944	0.885
	Circle	0.895	0.868	0.922	0.792	0.951	0.892
	Hybrid	0.912	0.888	0.935	0.824	0.954	0.909
	PF_FS	0.948	0.967	0.928	0.898	0.958	0.949
Independent test	FP4C	0.821	0.769	0.872	0.644	0.888	0.811
	CKDGraph	0.808	0.718	0.897	0.626	0.893	0.789
	AP2D	0.872	0.769	0.974	0.760	0.900	0.857
	Estate	0.769	0.744	0.795	0.539	0.890	0.763
	KR	0.859	0.744	0.974	0.738	0.931	0.841
	CKD	0.859	0.769	0.949	0.730	0.943	0.845
	CKDExt	0.859	0.795	0.923	0.724	0.937	0.849
	PubChem	0.833	0.821	0.846	0.667	0.914	0.831
	MACCS	0.846	0.795	0.897	0.696	0.922	0.838
	FP4	0.872	0.846	0.897	0.745	0.916	0.868
	Circle	0.859	0.744	0.974	0.738	0.935	0.841
	Hybrid	0.821	0.744	0.897	0.649	0.907	0.806
	PF_FS	0.897	0.846	0.949	0.799	0.938	0.892

**Fig. 4:**
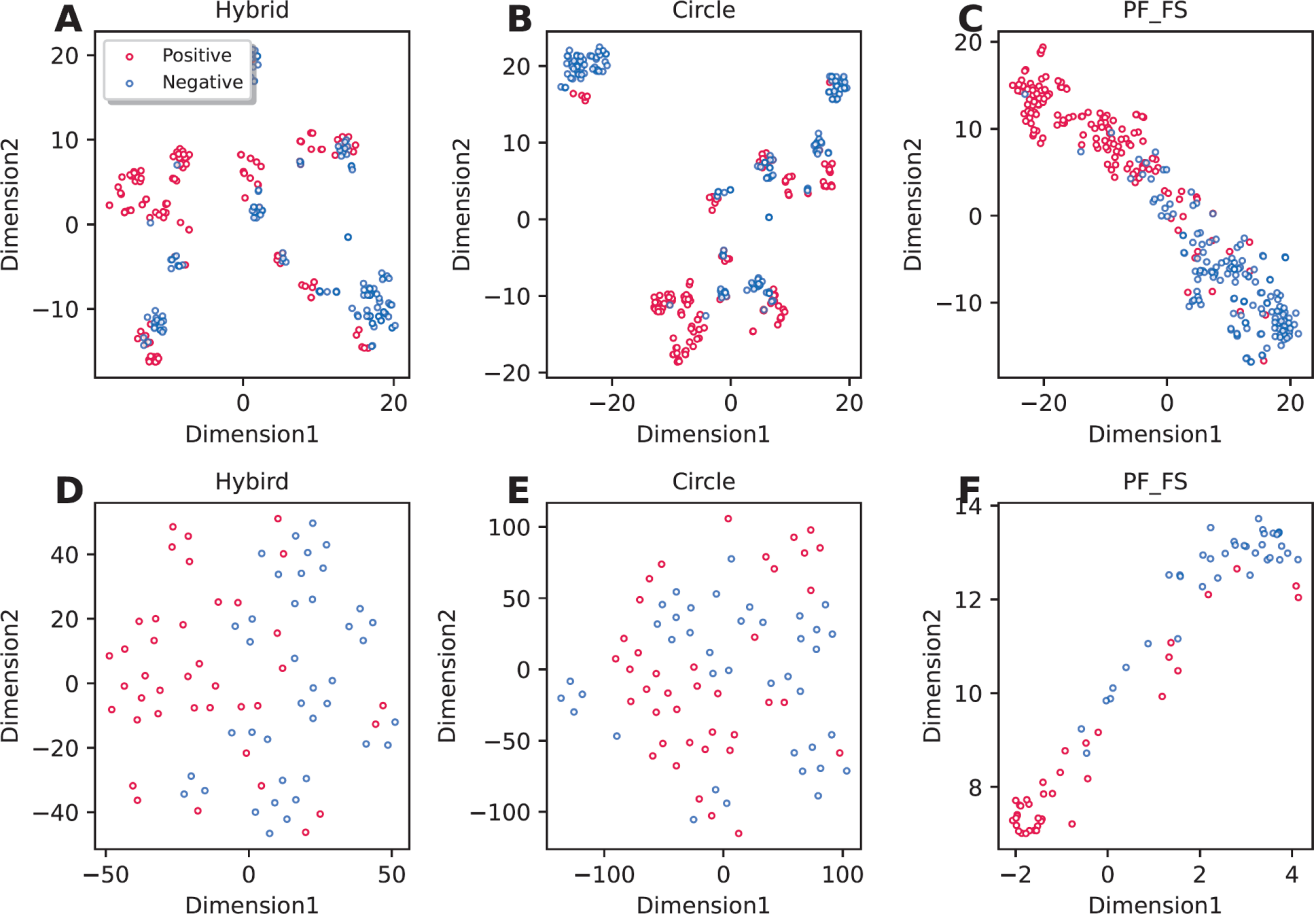
Visualization of t-SNE distribution for our generated feature PF_FS and two compared molecular descriptors (i.e., Hybrid and Circle) on the training (**A–C**) and independent test (**D-F**) datasets.

Furthermore, the highest MCC of 0.792 and 0.824 were achieved using Circle and Hybrid on the CGRP-TRN dataset, respectively. This indicates that Circle and Hybrid were the most powerful molecular descriptors in the identification of CGRP inhibitors. For the convenience of our investigation, we compared the performance of the PF_FS against these two molecular descriptors in terms of their predictive ability using tSNE approach (Fig. 4) and their overall performance (Table 4). From the comparison results summarized in Table 4, it is clear that the overall performance of the PF_FS is better than all the 12 conventional molecular descriptors in terms of the CGRP-TRN dataset. Furthermore, in case of the CGRP-IND dataset, the ACC, MCC, and F1 of the PF_FS were 3.85–7.69, 6.12–15.03, 5.13–8.63% higher than that of Circle and Hybrid, indicating the contribution of the PF_FS for performance improvement. Similarly, in Fig. 4, the positive and negative classes (i.e., active and inactive compounds) are clearly identifiable as two distinct clusters using our generated PF_FS feature, compared to Circle and Hybrid, for both the CGRP-TRN and CGRP-IND datasets. This demonstrates that the PF_FS feature generated in this study provides more significant information for improving CGRP inhibitor identification.

**Fig. 5:**
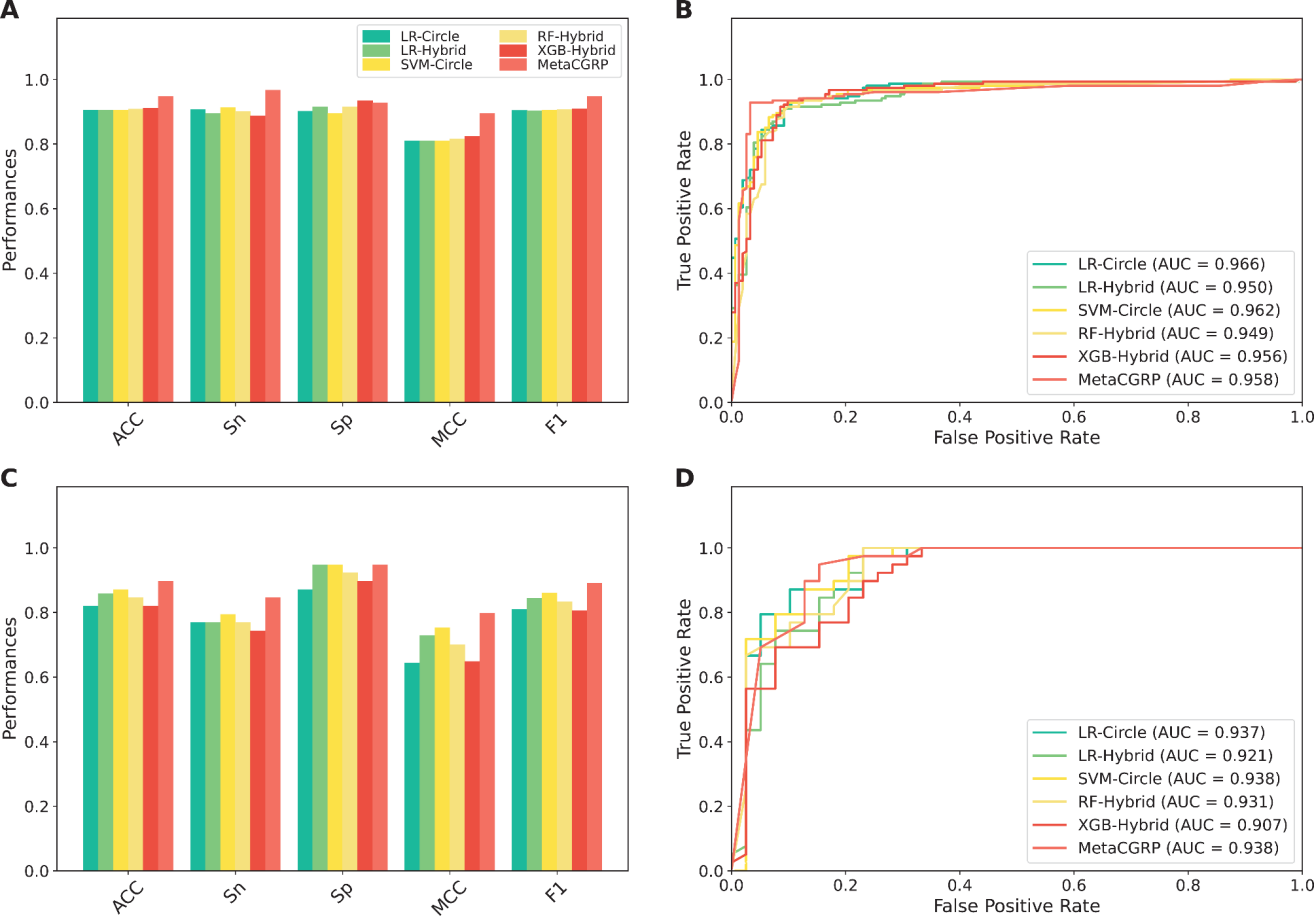
Performance comparison of our proposed MetaCGRP and top-five baseline models on the training (**A–B**) and independent test (**C–D**) datasets.

In the second comparative experiment, we compared the performance of MetaCGRP with the top-five powerful baseline models having the highest cross-validation MCC (i.e., LR-Circle, LR-Hybrid, SVM-Circle, RF-Hybrid, and XGB-Hybrid). Based on the same CGRP-TRN and CGRP-IND datasets, Fig. 5; Table 5 display the 10-fold cross-validation and independent test results of the proposed MetaCGRP with the top-five powerful prediction models. It can clearly be seen that MetaCGRP attained better performance than the top-five powerful baseline models as judged by four out of six metrics, including ACC, SN, F1, and MCC, on both the CGRP-TRN and CGRP-IND datasets. In case of the CGRP-IND dataset, the ACC, SN, F1, and MCC, and F1 of MetaCGRP were 2.56–7.69, 5.13–10.26, 30.8–8.11, 4.65–15.47% higher than that of the top-five powerful baseline models. In addition, to verify the stability of the proposed model, MetaCGRP was performed on 10 individual cross-validation and independent test results over corresponding training and independent test datasets. The detailed results are recorded in Supplementary Table S7. The average ± STD AUC scores of MetaCGRP were 0.952 ± 0.011 and 0.943 ± 0.016 over the training and independent test datasets, respectively. Taken together, these results reveal that MetaCGRP could efficiently attain more accurate and stable CGRP inhibitor identification in terms of both the 10-fold cross-validation and independent tests.

**Table 5:** Performance comparison of MetaCGRP and top-five baseline models on the training and independent test datasets.

Evaluation strategy	Method	ACC	SN	SP	MCC	AUC	F1
Cross-validation	LR-Circle	0.905	0.908	0.903	0.810	0.961	0.905
	LR-Hybrid	0.905	0.895	0.916	0.811	0.950	0.904
	SVM-Circle	0.905	0.914	0.896	0.811	0.956	0.906
	RF-Hybrid	0.908	0.901	0.916	0.817	0.944	0.907
	XGB-Hybrid	0.912	0.888	0.935	0.824	0.954	0.909
	MetaCGRP	0.948	0.967	0.928	0.898	0.958	0.949
Independent test	LR-Circle	0.821	0.769	0.872	0.644	0.937	0.811
	LR-Hybrid	0.859	0.769	0.949	0.730	0.921	0.845
	SVM-Circle	0.872	0.795	0.949	0.753	0.938	0.861
	RF-Hybrid	0.846	0.769	0.923	0.701	0.931	0.833
	XGB-Hybrid	0.821	0.744	0.897	0.649	0.907	0.806
	MetaCGRP	0.897	0.846	0.949	0.799	0.938	0.892

### Feature importance analysis

**Fig. 6:**
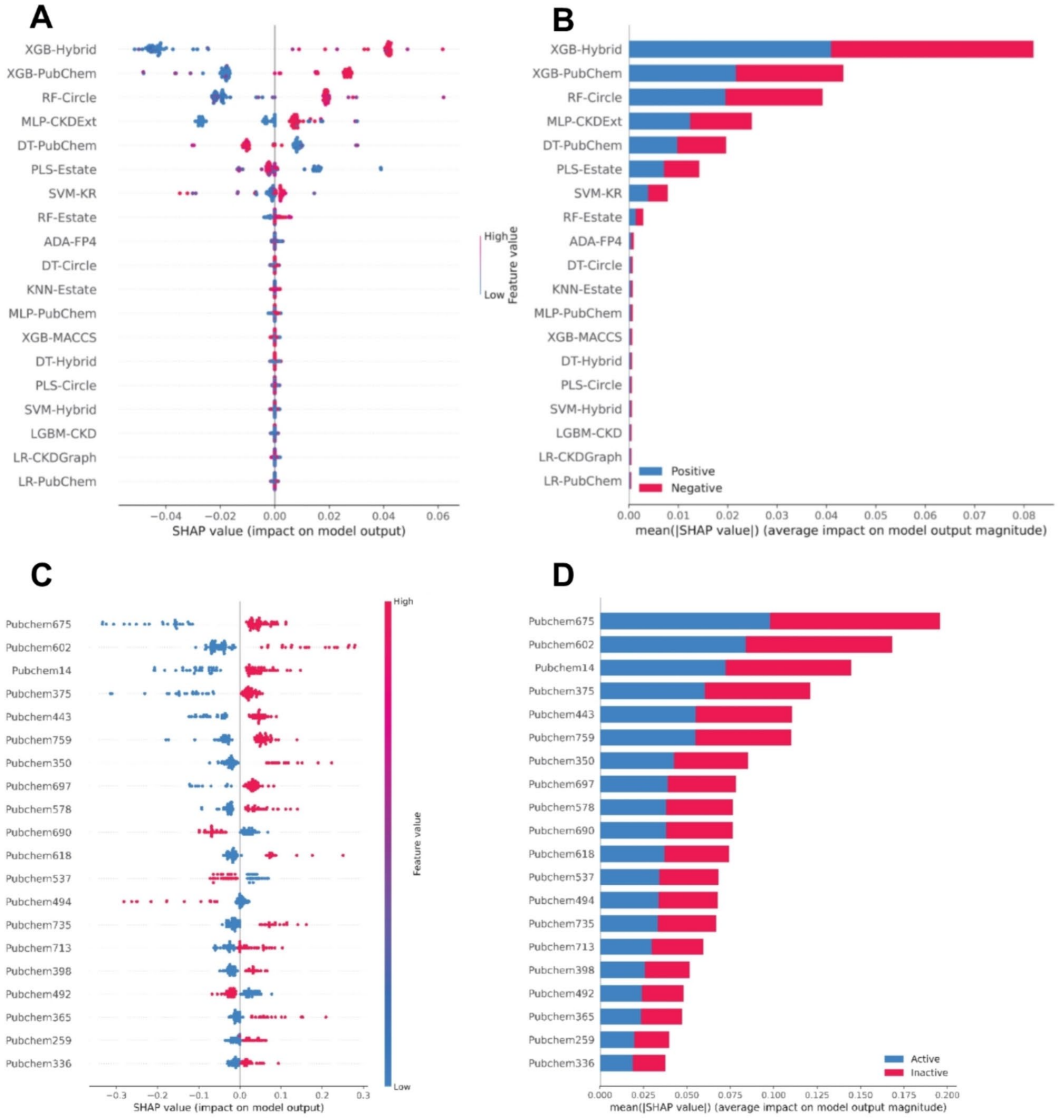
Feature importance from MetaCGRP **(A-B)** and XGB-PubChem **(C-D)** as ranked by SHAP values based on the training dataset. (**A** and **C**) Magnitude and direction of the contribution of each feature to the model prediction of CGRP inhibitors. (**B** and **D**) Mean absolute SHAP values, where positive and negatives SHAP values influences the predictions toward positive and negative samples, respectively.

In this section, we utilize the Shapley Additive explanation (SHAP) method^76^ to improve our understanding of MetaCGRP’s output and highlight the important features^48,49,51,52^. Features exhibiting a strongly positive value, indicated by the red color toward the positive x-axis, were considered highly influential in CGRP inhibition. As mentioned above, the PF_FS was generated based on 19 selected baseline models (Fig. 6A-B and Supplementary Table S8). It could be observed that the top-five informative features consisted of XGB-Hybrid, XGB-Pubchem, RF-Circle, MLP-CKDExt, and DT-Pubchem. Based on their SHAP values, almost all top-five informative PFs, with the only exception of DT-Pubchem, exhibited a strongly positive value, highlighting that these features were considered highly influential in CGRP inhibition. In additional, we utilize the SHAP method to analyze the XGB-Pubchem output in order to gain a deeper insight into the specific substructural elements that may be responsible for potential inhibitory effects against CGRP. Figure 6C-D and Supplementary Table S9 show the top 20 important features. The details of these analyzed substructure fragments, along with their SMARTS patterns, are presented in Table 6.

**Table 6:** Summary of the top-twenty important features ranked by SHAP along with their corresponding substructure descriptions and SMARTS pattern.

Rank	Feature	SMARTS pattern	Substructure description
1	Pubchem675	Br-C-C-C: C	1-Bromobutane
2	Pubchem602	O = C-C-N-C	2-(methylamino)acetaldehyde
3	Pubchem14	>= 1 N	Greater than or equal to 1 nitrogen atom
4	Pubchem375	C(~ N)(~ N)	Methanediamine
5	Pubchem443	C(-C)(= O)	acetaldehyde
6	Pubchem759	Cc1c(Cl)cccc1	2-Chlorotoluene
7	Pubchem350	C(~ C)(~ I)	Iodoethane
8	Pubchem697	C-C-C-C-C-C(C)-C	2-methyl-heptane
9	Pubchem578	C-C: C-C: C	Pentane
10	Pubchem690	O-C-C-C-C-C-O	1,5-Pentanediol
11	Pubchem618	C: C-C-C: C	Pentane
12	Pubchem537	O = C-C-O	Glycolaldehyde
13	Pubchem494	O = C-C: N	2-Aminoacetaldehyde
14	Pubchem735	Cc1cc(O)ccc1	3-methylphenol
15	Pubchem713	Cc1ccc(C)cc1	P-Xylene
16	Pubchem398	N(~ H)(~ N)	Hydrazine
17	Pubchem492	N-C: O:C	1-Methoxymethanamine
18	Pubchem365	C(~ H)(~ N)	Methylamine
19	Pubchem259	>= 3 aromatic rings	Greater than or equal to 3 aromatic rings
20	Pubchem336	C(~ C)(~ C)(~ C)(~ N)	2-methylpropan-2-amine

It is noteworthy that among the top 20 important substructures, 16 exhibit positive and high feature values (indicated by red on the positive scale in Fig. 6D), thereby contributing significantly to CGRP inhibition. Particularly remarkable is that six out of these 16 features consist of nitrogen-containing substructures, including 2-(methylamino) acetaldehyde, methanediamine, hydrazine, methylamine, and 2-methylpropan-2-amine. The substantial contribution from nitrogen-containing compounds in the top features is unsurprising, given their wide utilization in medicinal chemistry^77^. Notably, among these, 2-(methylamino) acetaldehyde (Pubchem602), methanediamine (Pubchem375), and methylamine (Pubchem365) stand out. These amides are characterized by a nitrogen atom directly bonded to carbon atoms and serve as key precursor elements in the manufacturing of pharmaceutical compounds due to their ability to form hydrogen bonds and interact with receptor sites, which are essential properties in drug design. Moreover, studies have shown that 2-(methylamino) acetaldehyde is a part of secondary amines and is associated with mu opioid antagonism (patent: US-7902221-B2), while methanediamine serves as a precursor for compounds exhibiting anti-cancer activities^78^. Additionally, nitrogen substructures play a significant role in CGRP inhibition, as evidenced by nitrogen-based heterocyclic substructures in FDA-approved (Supplementary Figure S4) and newly designed small molecule CGRP inhibitors^79,80^. These drugs utilize nitrogen-containing structures to enhance their binding affinity and specificity for CGRP receptors, thereby inhibiting CGRP signaling and providing relief from migraine symptoms. The role of nitrogen in these compounds highlights the importance of such functional groups in the development of effective CGRP inhibitors.

Furthermore, other substructures that make substantial contributions include halogen-containing ones, such as 1-bromobutane, 2-chlorotoluene, and iodoethane corresponding to Pubchem675, Pubchem759, and Pubchem350, respectively. Furthermore, alkanes (i.e., pentane and 2-methyl-heptane), acetaldehyde, 3-methylphenol, and p-xylene also play significant roles. Halogen motifs can influence the pharmacokinetic and pharmacodynamic properties of drugs, including enhanced binding affinity, metabolic stability, structure optimization, and bioisosteric replacement^81–83^. This influence is evident from Supplementary Figure S4, where three of the four FDA-approved drugs for CGRP contain a halogen motif that interacts with the CGRP receptor. Moreover, the most prominent feature was 1-bromobutane (Pubchem675), also known as butylbromide, which has been implicated in several studies regarding the inhibition of CGRP in irritable bowel syndrome^84,85^. However, no study using butylbromide has been conducted on the inhibition of CGRP in migraines. The incorporation of the aforementioned substructures into drug molecules can enhance various interactions, including hydrophobic, halogen bonding, and π-π stacking, all of which are critical for effective CGRP receptor binding and inhibition.

Furthermore, it is interesting to note that six of the 16 important features are natural products (Fig. 6D-C; Table 6). For example, Pubchem713, which pertains to p-xylene, is a natural product found in *Basella alba* (malabar spinach), *Helianthus tuberosus* (Jerusalem artichoke) among others^86^. Shanta et al.^87^ reported that the viscous liquid obtained from the leaves and tender stalks of *Basella alba* is a remedy for habitual headaches. Similarly, Sawicka et al.^88^ studied the beneficial effects of *Helianthus tuberosus* and found it can function as anti-diabetic, anti-carcinogenic, anti-fungistatic, anti-constipation, metabolism-improving, and body mass-reducing agents. However, their effects on migraine have not been studied. Likewise, iodoethane (Pubchem350), pentane (Pubchem578 and Pubchem618), and methylamine (Pubchem365) are all derived from natural products found in *Mastocarpus stellatus* and *Fucus vesiculosus*, *Calendula officinalis* and *Allium ampeloprasum*, and *Peucedanum palustre* and *Vitis vinifera*, respectively, which show positive feature contributions towards active compounds. The products obtained from some of the above-mentioned species have been indicated as migraine relief medications in alternative treatments such as homeopathy, ayurveda and TCM, as they all exhibit anti-oxidant and anti-inflammatory properties^89,90^. However, relatively little research indicates the anti-migraine potential of these natural products^91–94^. Collectively, the important features identified by SHAP analysis can potentially serve as active substructures for CGRP inhibitors.

### Discovery of new drugs to treat migraine through inhibition of CGRP using Thai Herbal Pharmacopoeia

**Fig. 7:**
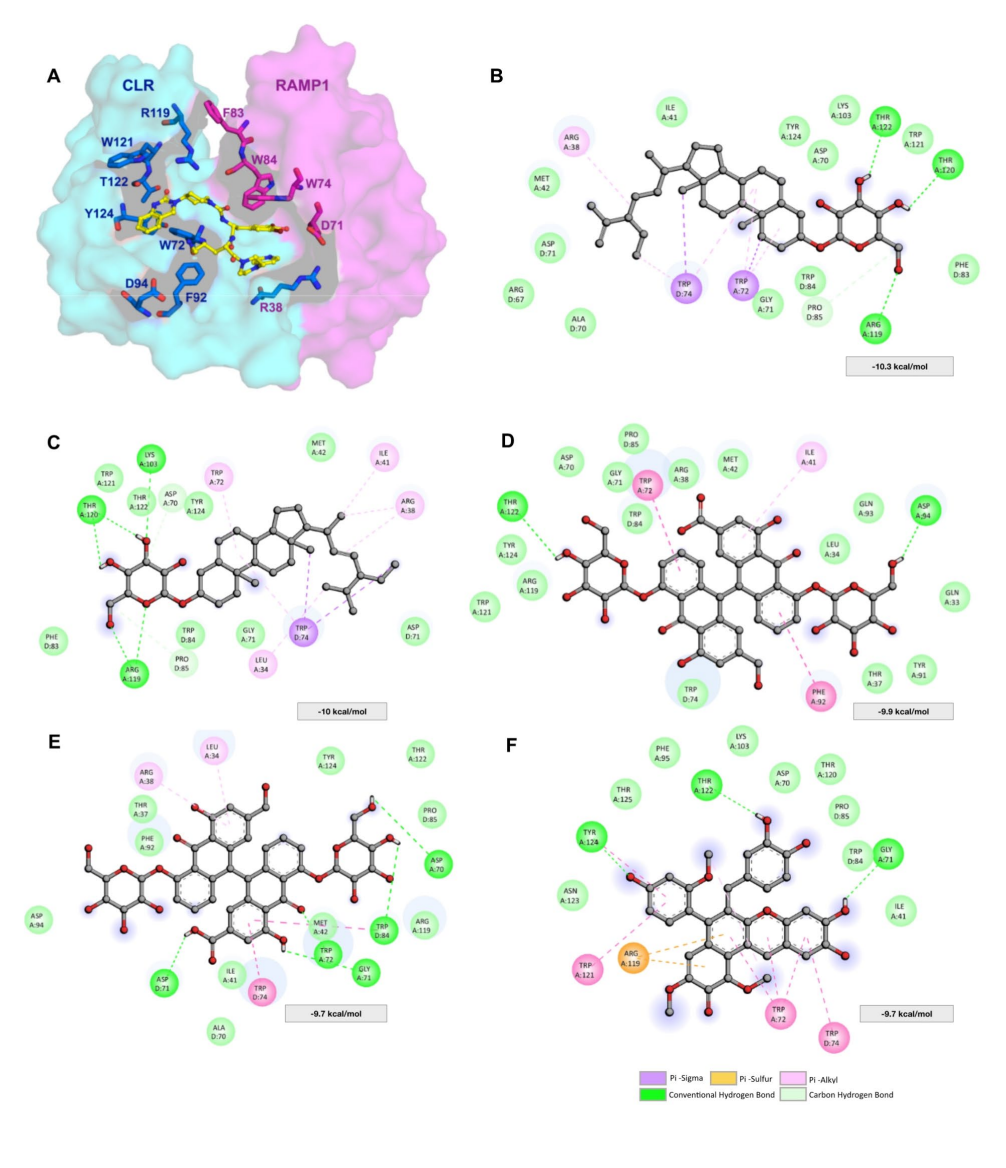
Protein-ligand interactions of CGRP (PDB ID: 3N7S) with **(A)** the co-crystal ligand (ocelgepant in yellow) where CLR and RAMP1 subunits are highlighted in cyan and magenta, respectively. Close-up views of the binding interaction of CGRP and **(B)** clerosterol 3-glucoside, **(C)** stigmasterol 3-glucoside, **(D)** sennoside D, **(E)** sennoside C, and **(F)** stantalin A.

In the preceding sections, we demonstrated that MetaCGRP outperformed several traditional ML classifiers, showcasing its superior performance. Therefore, in this section, we utilized MetaCGRP for virtual screening, employing data from the Thai herbal pharmacopoeia to identify potential natural compounds with activity against CGRP. In addition, we conducted molecular docking analyses to uncover how these compounds bind and to assess their binding affinities. For this investigation, we utilized the crystal structure of CGRP in complex with an inhibitor as a reference (further details in Material and Methods). Table 7 provides a list of FDA-approved CGRP inhibitors and the top-five natural compounds, along with their probabilities, corresponding docking scores, and interaction residues. Figure 7 illustrates the protein structure of CGRP in its docked conformation, highlighting the interacting residues of the top-five compounds. The docking scores for the top-five compounds were as follows: -10.3, -10, -9.9, -9.7, and − 9.7 kcal/mol corresponding to clerosterol 3-glucoside, stigmasterol 3-glucoside, sennoside D, sennoside C, and stantalin A, respectively. These scores were comparable to two FDA-approved drugs, namely ubrogepant, and rimegepant, with docking scores of -10.1 and − 9.8 kcal/mol, respectively (Table 7).

**Table 7:** List of FDA-approved CGRP inhibitors and the top-five natural compounds, along with their probability, corresponding docking scores and interaction residues.

Compound	Compound name	Probability	Docking score (kcal/mol)	Interacting residues
FDA-approved	Ubrogepant (December 2019)	0.562	− 10.1	**CLR**: Q33, L34, T37, I41, M42, G71, W72, F92, Q93, D94 **RAMP1**: A70, D71, W74, W84, P85
	Rimegepant (February 2020)	0.530	− 9.8	**CLR**: L34, T37, R38, I41, M42, D70, G71, W72, F92 **RAMP1**: A70, D71, R67, W74, W84, P85
	Zavegepant (March 2023)	0.530	− 12.7	**CLR**: L34, R38, I41, M42, D70, G71, W72, F92, R119, W121, T122, Y124, T125 **RAMP1**: A70, D71, W74, W84, P85
	Atogepant (April 2023)	0.587	− 11.2	**CLR**: L34, T37, R38, I41, M42, W72, F92, R119, T120, W121, T122, Y124 **RAMP1**: R67, A70, D71, W74, W84, P85
Co-crystal ligand	Olcegepant (PDB ID: 3N7S)	0.587	− 8.6	**CLR**: R38, W72, F92, D94, R119, W121, T122, Y124 **RAMP1**: D71, W74, F83, W84
Top 5 candidates	Clerosterol 3-glucoside	0.530	− 10.3	**CLR**: R38, I41, M42, D70, G71, W72, K103, R119, T120, W121, T122, Y124 **RAMP1**: R67, A70, D71, W74, F83, W84, P85
	Stigmasterol 3-glucoside	0.530	− 10	**CLR**: L34, R38, I41, M42, D70, G71, K103, R119, T120, W121, T122, Y124 **RAMP1**: D71, W74, F83, W84, P85
	Sennoside D	0.543	− 9.9	**CLR**: Q33, L34, T37, R38, I41, M42, D70, G71, W72, F92, Q93, D94, R119, W121, T122, Y124 **RAMP1**: W74, W84, P85
	Sennoside C	0.543	− 9.7	**CLR**: L34, T37, R38, I41, M42, D70, G71, W72, F92, D94, R119, T122, Y124 **RAMP1**: A70, D71, W74, W84, P85
	Stantalin A	0.530	− 9.7	**CLR**: I41, D70, G71, W72, F95, K103, R119, T120, W121, T122, N123, Y124, T125 **RAMP1**: W74, W84, P85

The top-two compounds (i.e., clerosterol 3-glucoside and stigmasterol 3-glucoside) are natural products found in various plants, including bamboo and rice bran^95^. They belong to the class of phytosterols, which are sterol compounds derived from plants. Extensive studies have been conducted on these compounds for their prospective health-promoting benefits, such as their anti-cancer and anti-inflammatory properties^96,97^. Hernández-Flores et al.^98^ studied the analgesic effects of phytosterol-derived ibuprofen-like substances while mitigating the gastric side effects, in comparison with ibuprofen, and found that the phytosteryl ibuprofenates (including stigmasteryl (S)-ibuprofenate and cholesteryl (S)-ibuprofenate, among others) possessed comparable activity to ibuprofen at the same mg/kg doses, but without the associated gastric effects. This analgesic effect could be beneficial in migraine treatment. Moreover, they have shown promise in the treatment of cardiovascular diseases due to their cholesterol-lowering effects^99,100^. Among the top-two compounds, more literature is present on the benefits of stigmasterol as it is among the most abundant sterols found in plants, with a major role in maintaining the structure and function of cell membranes^101^. In addition, stigmasterol was shown to exert anticonvulsant effects when assessed on various recombinant GABA_A_ receptor subtypes^102^. Furthermore, a study by Parthasarathy et al. 10.1016/j.fshw.2019.01.001 screened thirteen anti-migraine compounds from the leaves of *Abrus precatorius* (i.e., Indian liquorice) identified through GCMS analysis against CGRP. The authors identified stigmasterol, among the thirteen anti-migraine compounds through molecular docking that could serve as inhibitors for migraine headache.

Upon examining the binding interactions between the top compound (i.e., clerosterol 3-glucoside (Fig. 7B)) and the ectodomain complex of the CGRP receptor, H-bonds were observed with residues ARG119^CLR^, THR120^CLR^ and THR122^CLR^, along with π-sigma and π-alkyl interactions with residues ARG38^CLR^, TRP72^CLR^, and TRP74^RAMP1^. Additionally, hydrophobic interactions involved ILE41^CLR^, MET42^CLR^, ARG67^RAMP1^, ALA70^RAMP1^, ASP70^CLR^, ASP71^RAMP1^, GLY71^CLR^, PHE83^RAMP1^, TRP84^RAMP1^, PRO85^RAMP1^, LYS103^CLR^, TRP121^CLR^, and TYR124^CLR^. These interactions are consistent with those observed in FDA-approved CGRP antagonists (Supplementary Figure S4). Similarly, stigmasterol 3-glucoside forms H-bonds with ARG119^CLR^, THR120^CLR^, and LYS103^CLR^, along with π-sigma (TRP74^RAMP1^) and π-alkyl interactions (LEU34^CLR^, ARG38^CLR^, ILE41^CLR^, and TRP72^CLR^), consistent with clerosterol 3-glucoside. Moreover, some studies have suggested a correlation between high cholesterol levels and migraine^103^, thus the cholesterol-lowering effects of these compounds derived from natural products may have potential applications in migraine treatment.

Similarly, the other top compounds also demonstrated H-bonds and hydrophobic interactions with the residues mentioned above, albeit in different capacities (Fig. 7C-F; Table 7). Taking it a step further, we calculated the physicochemical properties of the top-five compounds and the FDA-approved drugs, presented in Supplementary Table S10. Clerosterol 3-glucoside, stigmasterol 3-glucoside and stantalin A demonstrated the closest adherence to the Ro5 and Veber’s rule for orally active drugs. However, sennoside D and sennoside C showed violations for all criteria except nRotB, indicating that they are not suitable for further development. Therefore, clerosterol 3-glucoside and stigmasterol 3-glucoside show the greatest promise and can be explored in future research endeavors.

## Limitations and future development

While the proposed MetaCGRP model demonstrates a promising performance in predicting CGRP inhibitors, several limitations and future research directions must be addressed to enhance its practical utility. First of all, we plan to collect additional active and inactive compounds, and then combine them into the existing dataset. Secondly, we will apply Mol2Vec^104^, which is an unsupervised pretraining method, to generate molecular embeddings. Recently, our research study showed that the combination of molecular fingerprints and molecular embeddings were capable of contributing to performance improvement^105^. Third, we can try to merge MetaCRGP with novel and powerful ML frameworks, such as contrastive learning^106,107^ and deep meta-learning method^108^, to alleviate the curse of limited samples. In addition, the model’s applicability to other drug targets remains uncertain, as it has been specifically optimized for CGRP-related compounds. Future work will involve validating the model with independent and diverse datasets, including those related to different peptide targets such as other neuropeptides (such as Substance P or Neurokinin A) implicated in pain pathways, to assess its generalizability and robustness across various therapeutic areas. Additionally, the current study does not fully explore the computational costs and scalability of the model, particularly when applied to large datasets in a real-world setting. We plan to explore more efficient training algorithms and cloud-based distributed computing to mitigate these challenges. Moreover, integrating MetaCGRP with other drug discovery tools, such as molecular docking software, is a priority for future development. This integration will enable the model to be part of a multi-tool approach for in silico screening platform, improving the efficiency and accuracy of the drug discovery processes. By addressing these areas, we aim to enhance the model’s broader applicability and ensure its successful deployment in real-world drug discovery pipelines.

## Conclusion

Computational approaches with the ability to precisely detect CGRP inhibitors hold significant potential for expediting the high-throughput discovery of new CGRP drugs. In this study, we propose MetaCGRP, a high-accuracy computation approach designed to identify CGRP inhibitors without the need for 3D structural information. MetaCGRP leverages multiple strengths derived from different molecular representation methods, coupled with popular ML algorithms, to construct a meta-learning predictor. To the best of our knowledge, MetaCGRP is the first SMILES-based meta-model developed for identifying CGRP inhibitors without relying on 3D structural information. Independent testing demonstrated the exceptional performance of MetaCGRP compared to its baseline models, achieving ACC of 0.897, AUC of 0.938, and MCC of 0.799. When compared with well-known molecular descriptors, our new feature representations effectively discriminated active compounds from inactive ones. Based on SHAP analysis results, we identified the top 20 important features for CGRP inhibitors, including nitrogen and halogen substituents, as well as those derived from natural products, which were the most significant. To maximize the utility of our proposed model, we employed MetaCGRP for virtual screening, utilizing data from the Thai herbal pharmacopoeia to identify potential natural compounds with activity against CGRP. Finally, we implemented a user-friendly web server for MetaCGRP (accessible at https://pmlabqsar.pythonanywhere.com/MetaCGRP). We anticipate that MetaCGRP, as a valuable computational approach, will expedite the discovery of novel CGRP inhibitors from a large number of uncharacterized compounds.

## Electronic supplementary material

Below is the link to the electronic supplementary material.

Supplementary Material 1

## Data Availability

All data used in this study are available at https://pmlabqsar.pythonanywhere.com/MetaCGRP.
