# Supplementary material for: MetaCGRP is a high-precision meta-model for large-scale identification of CGRP inhibitors using multi-view information
Source: Sci Rep. 2024 Oct 21;14:24764. doi: 10.1038/s41598-024-75487-x (PMC11494111; doi:10.1038/s41598-024-75487-x)
Supplement: Supplementary file 1 — Supplementary Material 1 [file 41598_2024_75487_MOESM1_ESM.docx]

### Performance evaluation

The performance of baseline models and meta-models was six different performance measures, involving ACC, AUC, MCC, F1, SN, and SP. These measures are defined as follows.

| $SN=\frac{\mathrm{TP}}{\left( TP+FN \right)}$ | (1) |
| --- | --- |
| $SP=\frac{\mathrm{TN}}{\left( TN+FP \right)}$ | (2) |
| $ACC=\frac{TP+TN}{\left( TP+TN+FP+FN \right)}$ | (3) |
| $MCC=\frac{TP\times TN-FP\times FN}{\sqrt{(TP+FP)(TP+FN)(TN+FP)(TN+FN)}}$ | (4) |
| $F1=2\times\frac{TP}{2TP+FP+FN}$ | (5) |

where the numbers of correctly predicted positive and negative samples were referred to as TP and TN, respectively. On the other hand, the numbers of falsely predicted positive and negative samples are referred to as FP and FN, respectively.

## **Supplementary Figure**

## **Figure S1** Chemical space analysis of CGRP inhibitor dataset which include **(A)** molecular weight (MW), **(B)** Ghose-Crippen-Viswanadhan octanol-water partition coefficient (ALogP), **(C)** hydrogen bond acceptor (HBAc), **(D)** hydrogen bond donor (HBDon), **(E)** topological surface polar area (TPSA), and **(F)** number of rotatable bonds (nRotB). These descriptors differentiate between active and inactive chemical compounds, represented by green and pink colors, respectively.

**Figure S2** Chemical diversity analysis of CGRP inhibitor dataset depicted by Tanimoto similarity heatmap where high similarity values are indicated by orange and red colors, while low similarity values are presented by blue color.


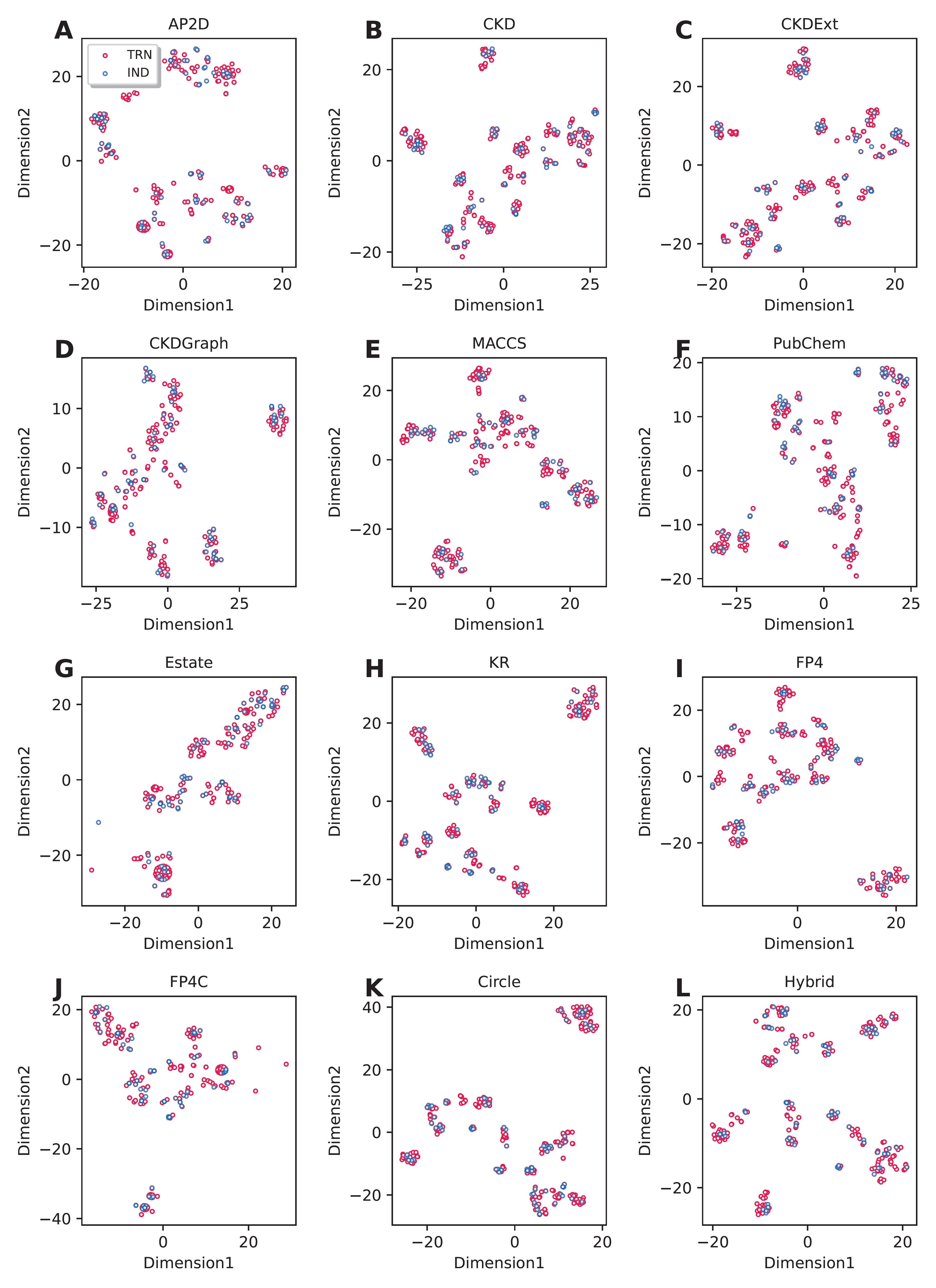


## **Figure S3** Chemical distribution of 12 molecular descriptors displayed by the t-Distributed Stochastic Neighbor Embedding (t-SNE) method, where red and blue dots indicate the training and independent test datasets, respectively.

## **Figure S4** Protein-ligand interactions of CGRP (PDB ID: 3N7S) and FDA-approved small molecule drugs where **(A)** Ubrogepant, **(B)** Rimegepant, **(C)** Zavegepant, and **(D)** Atogepant.

## **Supplementary Table**

## **Table S1** Cross-validation results of 144 baseline models developed using 12 different ML algorithms and 12 molecular descriptors.

| **Descriptor** | **Method** | **ACC** | **SN** | **SP** | **MCC** | **AUC** | **F1** |
| --- | --- | --- | --- | --- | --- | --- | --- |
| AP2D | ADA | 0.824 | 0.842 | 0.805 | 0.648 | 0.875 | 0.826 |
|  | DT | 0.830 | 0.816 | 0.844 | 0.660 | 0.862 | 0.827 |
|  | ET | 0.833 | 0.822 | 0.844 | 0.667 | 0.899 | 0.831 |
|  | KNN | 0.804 | 0.789 | 0.818 | 0.608 | 0.872 | 0.800 |
|  | LGBM | 0.837 | 0.842 | 0.831 | 0.673 | 0.915 | 0.837 |
|  | LR | 0.837 | 0.836 | 0.838 | 0.673 | 0.899 | 0.836 |
|  | MLP | 0.824 | 0.849 | 0.799 | 0.648 | 0.877 | 0.827 |
|  | NB | 0.683 | 0.539 | 0.825 | 0.380 | 0.798 | 0.628 |
|  | PLS | 0.771 | 0.789 | 0.753 | 0.543 | 0.861 | 0.774 |
|  | RF | 0.840 | 0.842 | 0.838 | 0.680 | 0.918 | 0.839 |
|  | SVM | 0.843 | 0.842 | 0.844 | 0.686 | 0.888 | 0.842 |
|  | XGB | 0.846 | 0.855 | 0.838 | 0.693 | 0.922 | 0.847 |
| Circle | ADA | 0.853 | 0.842 | 0.864 | 0.706 | 0.923 | 0.850 |
|  | DT | 0.843 | 0.829 | 0.857 | 0.686 | 0.879 | 0.840 |
|  | ET | 0.882 | 0.862 | 0.903 | 0.765 | 0.950 | 0.879 |
|  | KNN | 0.899 | 0.901 | 0.896 | 0.797 | 0.935 | 0.898 |
|  | LGBM | 0.886 | 0.875 | 0.896 | 0.771 | 0.949 | 0.884 |
|  | LR | 0.905 | 0.908 | 0.903 | 0.810 | 0.961 | 0.905 |
|  | MLP | 0.882 | 0.901 | 0.864 | 0.765 | 0.951 | 0.884 |
|  | NB | 0.850 | 0.855 | 0.844 | 0.699 | 0.866 | 0.850 |
|  | PLS | 0.830 | 0.809 | 0.851 | 0.661 | 0.929 | 0.826 |
|  | RF | 0.879 | 0.875 | 0.883 | 0.758 | 0.953 | 0.878 |
|  | SVM | 0.905 | 0.914 | 0.896 | 0.811 | 0.956 | 0.906 |
|  | XGB | 0.895 | 0.868 | 0.922 | 0.792 | 0.951 | 0.892 |
| CDK | ADA | 0.833 | 0.796 | 0.870 | 0.668 | 0.898 | 0.826 |
|  | DT | 0.863 | 0.842 | 0.883 | 0.726 | 0.869 | 0.859 |
|  | ET | 0.882 | 0.862 | 0.903 | 0.765 | 0.933 | 0.879 |
|  | KNN | 0.879 | 0.862 | 0.896 | 0.759 | 0.879 | 0.876 |
|  | LGBM | 0.859 | 0.836 | 0.883 | 0.720 | 0.932 | 0.855 |
|  | LR | 0.892 | 0.882 | 0.903 | 0.784 | 0.949 | 0.890 |
|  | MLP | 0.873 | 0.875 | 0.870 | 0.745 | 0.938 | 0.872 |
|  | NB | 0.840 | 0.776 | 0.903 | 0.685 | 0.847 | 0.828 |
|  | PLS | 0.863 | 0.882 | 0.844 | 0.726 | 0.923 | 0.865 |
|  | RF | 0.882 | 0.862 | 0.903 | 0.765 | 0.943 | 0.879 |
|  | SVM | 0.876 | 0.862 | 0.890 | 0.752 | 0.934 | 0.873 |
|  | XGB | 0.866 | 0.862 | 0.870 | 0.732 | 0.943 | 0.865 |
| CDKExt | ADA | 0.853 | 0.842 | 0.864 | 0.706 | 0.905 | 0.850 |
|  | DT | 0.850 | 0.829 | 0.870 | 0.700 | 0.922 | 0.846 |
|  | ET | 0.879 | 0.862 | 0.896 | 0.759 | 0.931 | 0.876 |
|  | KNN | 0.882 | 0.875 | 0.890 | 0.765 | 0.882 | 0.881 |
|  | LGBM | 0.866 | 0.855 | 0.877 | 0.732 | 0.934 | 0.864 |
|  | LR | 0.886 | 0.882 | 0.890 | 0.771 | 0.954 | 0.884 |
|  | MLP | 0.869 | 0.862 | 0.877 | 0.739 | 0.946 | 0.868 |
|  | NB | 0.843 | 0.803 | 0.883 | 0.688 | 0.853 | 0.836 |
|  | PLS | 0.853 | 0.862 | 0.844 | 0.706 | 0.925 | 0.853 |
|  | RF | 0.876 | 0.862 | 0.890 | 0.752 | 0.944 | 0.873 |
|  | SVM | 0.879 | 0.862 | 0.896 | 0.759 | 0.937 | 0.876 |
|  | XGB | 0.869 | 0.862 | 0.877 | 0.739 | 0.950 | 0.868 |
| CDKGraph | ADA | 0.840 | 0.855 | 0.825 | 0.680 | 0.884 | 0.841 |
|  | DT | 0.814 | 0.796 | 0.831 | 0.628 | 0.880 | 0.809 |
|  | ET | 0.859 | 0.836 | 0.883 | 0.720 | 0.910 | 0.855 |
|  | KNN | 0.814 | 0.750 | 0.877 | 0.632 | 0.871 | 0.800 |
|  | LGBM | 0.843 | 0.829 | 0.857 | 0.686 | 0.916 | 0.840 |
|  | LR | 0.866 | 0.849 | 0.883 | 0.732 | 0.915 | 0.863 |
|  | MLP | 0.843 | 0.822 | 0.864 | 0.687 | 0.899 | 0.839 |
|  | NB | 0.761 | 0.579 | 0.942 | 0.559 | 0.779 | 0.707 |
|  | PLS | 0.784 | 0.711 | 0.857 | 0.574 | 0.891 | 0.766 |
|  | RF | 0.850 | 0.836 | 0.864 | 0.700 | 0.920 | 0.847 |
|  | SVM | 0.856 | 0.855 | 0.857 | 0.712 | 0.901 | 0.855 |
|  | XGB | 0.840 | 0.836 | 0.844 | 0.680 | 0.923 | 0.838 |
| Estate | ADA | 0.729 | 0.730 | 0.727 | 0.458 | 0.825 | 0.728 |
|  | DT | 0.837 | 0.822 | 0.851 | 0.673 | 0.868 | 0.833 |
|  | ET | 0.833 | 0.816 | 0.851 | 0.667 | 0.879 | 0.829 |
|  | KNN | 0.807 | 0.842 | 0.773 | 0.616 | 0.874 | 0.813 |
|  | LGBM | 0.817 | 0.849 | 0.786 | 0.635 | 0.885 | 0.822 |
|  | LR | 0.765 | 0.737 | 0.792 | 0.530 | 0.845 | 0.757 |
|  | MLP | 0.807 | 0.836 | 0.779 | 0.616 | 0.866 | 0.812 |
|  | NB | 0.745 | 0.711 | 0.779 | 0.491 | 0.812 | 0.735 |
|  | PLS | 0.732 | 0.730 | 0.734 | 0.464 | 0.823 | 0.730 |
|  | RF | 0.837 | 0.849 | 0.825 | 0.673 | 0.905 | 0.838 |
|  | SVM | 0.837 | 0.868 | 0.805 | 0.675 | 0.863 | 0.841 |
|  | XGB | 0.850 | 0.868 | 0.831 | 0.700 | 0.906 | 0.852 |
| FP4 | ADA | 0.850 | 0.849 | 0.851 | 0.699 | 0.858 | 0.849 |
|  | DT | 0.850 | 0.796 | 0.903 | 0.703 | 0.899 | 0.840 |
|  | ET | 0.882 | 0.868 | 0.896 | 0.765 | 0.916 | 0.880 |
|  | KNN | 0.876 | 0.882 | 0.870 | 0.752 | 0.932 | 0.876 |
|  | LGBM | 0.886 | 0.888 | 0.883 | 0.771 | 0.939 | 0.885 |
|  | LR | 0.882 | 0.895 | 0.870 | 0.765 | 0.935 | 0.883 |
|  | MLP | 0.869 | 0.882 | 0.857 | 0.739 | 0.916 | 0.870 |
|  | NB | 0.752 | 0.921 | 0.584 | 0.536 | 0.848 | 0.787 |
|  | PLS | 0.801 | 0.829 | 0.773 | 0.602 | 0.890 | 0.805 |
|  | RF | 0.895 | 0.888 | 0.903 | 0.791 | 0.936 | 0.894 |
|  | SVM | 0.886 | 0.888 | 0.883 | 0.771 | 0.927 | 0.885 |
|  | XGB | 0.886 | 0.888 | 0.883 | 0.771 | 0.944 | 0.885 |
| FP4C | ADA | 0.824 | 0.868 | 0.779 | 0.650 | 0.854 | 0.830 |
|  | DT | 0.850 | 0.836 | 0.864 | 0.700 | 0.888 | 0.847 |
|  | ET | 0.859 | 0.855 | 0.864 | 0.719 | 0.919 | 0.858 |
|  | KNN | 0.817 | 0.829 | 0.805 | 0.634 | 0.867 | 0.818 |
|  | LGBM | 0.820 | 0.809 | 0.831 | 0.641 | 0.841 | 0.817 |
|  | LR | 0.843 | 0.868 | 0.818 | 0.687 | 0.898 | 0.846 |
|  | MLP | 0.853 | 0.849 | 0.857 | 0.706 | 0.906 | 0.851 |
|  | NB | 0.732 | 0.612 | 0.851 | 0.477 | 0.854 | 0.694 |
|  | PLS | 0.784 | 0.849 | 0.721 | 0.574 | 0.846 | 0.796 |
|  | RF | 0.869 | 0.862 | 0.877 | 0.739 | 0.923 | 0.868 |
|  | SVM | 0.863 | 0.862 | 0.864 | 0.725 | 0.892 | 0.862 |
|  | XGB | 0.830 | 0.829 | 0.831 | 0.660 | 0.911 | 0.829 |
| Hybrid | ADA | 0.869 | 0.855 | 0.883 | 0.739 | 0.932 | 0.867 |
|  | DT | 0.882 | 0.875 | 0.890 | 0.765 | 0.888 | 0.881 |
|  | ET | 0.892 | 0.888 | 0.896 | 0.784 | 0.935 | 0.891 |
|  | KNN | 0.886 | 0.882 | 0.890 | 0.771 | 0.886 | 0.884 |
|  | LGBM | 0.899 | 0.914 | 0.883 | 0.798 | 0.949 | 0.900 |
|  | LR | 0.905 | 0.895 | 0.916 | 0.811 | 0.950 | 0.904 |
|  | MLP | 0.879 | 0.882 | 0.877 | 0.758 | 0.938 | 0.879 |
|  | NB | 0.859 | 0.829 | 0.890 | 0.720 | 0.863 | 0.854 |
|  | PLS | 0.869 | 0.816 | 0.922 | 0.742 | 0.935 | 0.861 |
|  | RF | 0.908 | 0.901 | 0.916 | 0.817 | 0.944 | 0.907 |
|  | SVM | 0.895 | 0.888 | 0.903 | 0.791 | 0.937 | 0.894 |
|  | XGB | 0.912 | 0.888 | 0.935 | 0.824 | 0.954 | 0.909 |
| KR | ADA | 0.850 | 0.836 | 0.864 | 0.700 | 0.900 | 0.847 |
|  | DT | 0.869 | 0.882 | 0.857 | 0.739 | 0.878 | 0.870 |
|  | ET | 0.879 | 0.895 | 0.864 | 0.759 | 0.908 | 0.880 |
|  | KNN | 0.879 | 0.921 | 0.838 | 0.761 | 0.879 | 0.883 |
|  | LGBM | 0.876 | 0.875 | 0.877 | 0.752 | 0.943 | 0.875 |
|  | LR | 0.846 | 0.862 | 0.831 | 0.693 | 0.927 | 0.848 |
|  | MLP | 0.843 | 0.842 | 0.844 | 0.686 | 0.915 | 0.842 |
|  | NB | 0.771 | 0.855 | 0.688 | 0.551 | 0.777 | 0.788 |
|  | PLS | 0.820 | 0.796 | 0.844 | 0.641 | 0.891 | 0.815 |
|  | RF | 0.879 | 0.895 | 0.864 | 0.759 | 0.930 | 0.880 |
|  | SVM | 0.873 | 0.888 | 0.857 | 0.746 | 0.920 | 0.874 |
|  | XGB | 0.856 | 0.882 | 0.831 | 0.713 | 0.938 | 0.859 |
| MACCS | ADA | 0.859 | 0.862 | 0.857 | 0.719 | 0.907 | 0.859 |
|  | DT | 0.846 | 0.862 | 0.831 | 0.693 | 0.854 | 0.848 |
|  | ET | 0.882 | 0.875 | 0.890 | 0.765 | 0.914 | 0.881 |
|  | KNN | 0.876 | 0.914 | 0.838 | 0.754 | 0.916 | 0.880 |
|  | LGBM | 0.859 | 0.882 | 0.838 | 0.720 | 0.935 | 0.862 |
|  | LR | 0.876 | 0.875 | 0.877 | 0.752 | 0.924 | 0.875 |
|  | MLP | 0.863 | 0.882 | 0.844 | 0.726 | 0.916 | 0.865 |
|  | NB | 0.703 | 0.829 | 0.578 | 0.420 | 0.824 | 0.735 |
|  | PLS | 0.797 | 0.704 | 0.890 | 0.605 | 0.864 | 0.775 |
|  | RF | 0.873 | 0.875 | 0.870 | 0.745 | 0.935 | 0.872 |
|  | SVM | 0.869 | 0.868 | 0.870 | 0.739 | 0.920 | 0.868 |
|  | XGB | 0.879 | 0.882 | 0.877 | 0.758 | 0.938 | 0.879 |
| PubChem | ADA | 0.856 | 0.829 | 0.883 | 0.713 | 0.930 | 0.851 |
|  | DT | 0.833 | 0.803 | 0.864 | 0.668 | 0.883 | 0.827 |
|  | ET | 0.863 | 0.836 | 0.890 | 0.726 | 0.920 | 0.858 |
|  | KNN | 0.873 | 0.868 | 0.877 | 0.745 | 0.873 | 0.871 |
|  | LGBM | 0.876 | 0.888 | 0.864 | 0.752 | 0.944 | 0.877 |
|  | LR | 0.876 | 0.875 | 0.877 | 0.752 | 0.940 | 0.875 |
|  | MLP | 0.856 | 0.862 | 0.851 | 0.712 | 0.931 | 0.856 |
|  | NB | 0.735 | 0.763 | 0.708 | 0.472 | 0.762 | 0.741 |
|  | PLS | 0.775 | 0.658 | 0.890 | 0.563 | 0.896 | 0.743 |
|  | RF | 0.853 | 0.836 | 0.870 | 0.706 | 0.939 | 0.849 |
|  | SVM | 0.866 | 0.868 | 0.864 | 0.732 | 0.925 | 0.866 |
|  | XGB | 0.879 | 0.875 | 0.883 | 0.758 | 0.946 | 0.878 |

## **Table S2** Independent test results of 144 baseline models developed using 12 different ML algorithms and 12 molecular descriptors.

| **Descriptor** | **Method** | **ACC** | **SN** | **SP** | **MCC** | **AUC** | **F1** |
| --- | --- | --- | --- | --- | --- | --- | --- |
| AP2D | ADA | 0.846 | 0.718 | 0.974 | 0.716 | 0.880 | 0.824 |
|  | DT | 0.833 | 0.718 | 0.949 | 0.685 | 0.836 | 0.812 |
|  | ET | 0.833 | 0.795 | 0.872 | 0.669 | 0.907 | 0.827 |
|  | KNN | 0.808 | 0.846 | 0.769 | 0.617 | 0.876 | 0.815 |
|  | LGBM | 0.872 | 0.769 | 0.974 | 0.760 | 0.904 | 0.857 |
|  | LR | 0.821 | 0.692 | 0.949 | 0.663 | 0.852 | 0.794 |
|  | MLP | 0.808 | 0.769 | 0.846 | 0.617 | 0.873 | 0.800 |
|  | NB | 0.718 | 0.513 | 0.923 | 0.478 | 0.811 | 0.645 |
|  | PLS | 0.769 | 0.846 | 0.692 | 0.545 | 0.850 | 0.786 |
|  | RF | 0.872 | 0.821 | 0.923 | 0.748 | 0.908 | 0.865 |
|  | SVM | 0.833 | 0.769 | 0.897 | 0.672 | 0.872 | 0.822 |
|  | XGB | 0.872 | 0.769 | 0.974 | 0.760 | 0.900 | 0.857 |
| Circle | ADA | 0.821 | 0.744 | 0.897 | 0.649 | 0.904 | 0.806 |
|  | DT | 0.821 | 0.769 | 0.872 | 0.644 | 0.870 | 0.811 |
|  | ET | 0.846 | 0.769 | 0.923 | 0.701 | 0.943 | 0.833 |
|  | KNN | 0.872 | 0.795 | 0.949 | 0.753 | 0.945 | 0.861 |
|  | LGBM | 0.846 | 0.744 | 0.949 | 0.707 | 0.932 | 0.829 |
|  | LR | 0.821 | 0.769 | 0.872 | 0.644 | 0.937 | 0.811 |
|  | MLP | 0.833 | 0.795 | 0.872 | 0.669 | 0.927 | 0.827 |
|  | NB | 0.833 | 0.821 | 0.846 | 0.667 | 0.869 | 0.831 |
|  | PLS | 0.872 | 0.795 | 0.949 | 0.753 | 0.915 | 0.861 |
|  | RF | 0.872 | 0.769 | 0.974 | 0.760 | 0.945 | 0.857 |
|  | SVM | 0.872 | 0.795 | 0.949 | 0.753 | 0.938 | 0.861 |
|  | XGB | 0.859 | 0.744 | 0.974 | 0.738 | 0.935 | 0.841 |
| CDK | ADA | 0.872 | 0.795 | 0.949 | 0.753 | 0.936 | 0.861 |
|  | DT | 0.833 | 0.769 | 0.897 | 0.672 | 0.840 | 0.822 |
|  | ET | 0.808 | 0.744 | 0.872 | 0.621 | 0.924 | 0.795 |
|  | KNN | 0.821 | 0.744 | 0.897 | 0.649 | 0.821 | 0.806 |
|  | LGBM | 0.846 | 0.769 | 0.923 | 0.701 | 0.920 | 0.833 |
|  | LR | 0.897 | 0.846 | 0.949 | 0.799 | 0.936 | 0.892 |
|  | MLP | 0.846 | 0.821 | 0.872 | 0.693 | 0.933 | 0.842 |
|  | NB | 0.872 | 0.769 | 0.974 | 0.760 | 0.906 | 0.857 |
|  | PLS | 0.846 | 0.846 | 0.846 | 0.692 | 0.917 | 0.846 |
|  | RF | 0.859 | 0.744 | 0.974 | 0.738 | 0.936 | 0.841 |
|  | SVM | 0.846 | 0.769 | 0.923 | 0.701 | 0.944 | 0.833 |
|  | XGB | 0.859 | 0.769 | 0.949 | 0.730 | 0.943 | 0.845 |
| CDKExt | ADA | 0.859 | 0.821 | 0.897 | 0.720 | 0.909 | 0.853 |
|  | DT | 0.795 | 0.846 | 0.744 | 0.593 | 0.893 | 0.805 |
|  | ET | 0.833 | 0.795 | 0.872 | 0.669 | 0.912 | 0.827 |
|  | KNN | 0.833 | 0.769 | 0.897 | 0.672 | 0.833 | 0.822 |
|  | LGBM | 0.821 | 0.744 | 0.897 | 0.649 | 0.918 | 0.806 |
|  | LR | 0.859 | 0.821 | 0.897 | 0.720 | 0.932 | 0.853 |
|  | MLP | 0.846 | 0.769 | 0.923 | 0.701 | 0.931 | 0.833 |
|  | NB | 0.872 | 0.769 | 0.974 | 0.760 | 0.894 | 0.857 |
|  | PLS | 0.821 | 0.821 | 0.821 | 0.641 | 0.913 | 0.821 |
|  | RF | 0.859 | 0.769 | 0.949 | 0.730 | 0.946 | 0.845 |
|  | SVM | 0.846 | 0.769 | 0.923 | 0.701 | 0.935 | 0.833 |
|  | XGB | 0.859 | 0.795 | 0.923 | 0.724 | 0.937 | 0.849 |
| CDKGraph | ADA | 0.769 | 0.744 | 0.795 | 0.539 | 0.808 | 0.763 |
|  | DT | 0.833 | 0.769 | 0.897 | 0.672 | 0.858 | 0.822 |
|  | ET | 0.795 | 0.692 | 0.897 | 0.603 | 0.911 | 0.771 |
|  | KNN | 0.846 | 0.744 | 0.949 | 0.707 | 0.877 | 0.829 |
|  | LGBM | 0.795 | 0.718 | 0.872 | 0.597 | 0.902 | 0.778 |
|  | LR | 0.795 | 0.744 | 0.846 | 0.593 | 0.882 | 0.784 |
|  | MLP | 0.808 | 0.692 | 0.923 | 0.632 | 0.862 | 0.783 |
|  | NB | 0.782 | 0.641 | 0.923 | 0.588 | 0.777 | 0.746 |
|  | PLS | 0.846 | 0.872 | 0.821 | 0.693 | 0.891 | 0.850 |
|  | RF | 0.833 | 0.744 | 0.923 | 0.678 | 0.919 | 0.817 |
|  | SVM | 0.808 | 0.769 | 0.846 | 0.617 | 0.869 | 0.800 |
|  | XGB | 0.808 | 0.718 | 0.897 | 0.626 | 0.893 | 0.789 |
| Estate | ADA | 0.705 | 0.692 | 0.718 | 0.410 | 0.813 | 0.701 |
|  | DT | 0.769 | 0.769 | 0.769 | 0.538 | 0.863 | 0.769 |
|  | ET | 0.769 | 0.744 | 0.795 | 0.539 | 0.851 | 0.763 |
|  | KNN | 0.769 | 0.718 | 0.821 | 0.541 | 0.870 | 0.757 |
|  | LGBM | 0.795 | 0.769 | 0.821 | 0.591 | 0.887 | 0.789 |
|  | LR | 0.705 | 0.692 | 0.718 | 0.410 | 0.795 | 0.701 |
|  | MLP | 0.795 | 0.821 | 0.769 | 0.591 | 0.912 | 0.800 |
|  | NB | 0.654 | 0.692 | 0.615 | 0.309 | 0.749 | 0.667 |
|  | PLS | 0.667 | 0.718 | 0.615 | 0.335 | 0.797 | 0.683 |
|  | RF | 0.769 | 0.769 | 0.769 | 0.538 | 0.878 | 0.769 |
|  | SVM | 0.795 | 0.769 | 0.821 | 0.591 | 0.867 | 0.789 |
|  | XGB | 0.769 | 0.744 | 0.795 | 0.539 | 0.890 | 0.763 |
| FP4 | ADA | 0.821 | 0.769 | 0.872 | 0.644 | 0.909 | 0.811 |
|  | DT | 0.821 | 0.718 | 0.923 | 0.655 | 0.886 | 0.800 |
|  | ET | 0.833 | 0.744 | 0.923 | 0.678 | 0.884 | 0.817 |
|  | KNN | 0.833 | 0.821 | 0.846 | 0.667 | 0.891 | 0.831 |
|  | LGBM | 0.859 | 0.821 | 0.897 | 0.720 | 0.921 | 0.853 |
|  | LR | 0.833 | 0.795 | 0.872 | 0.669 | 0.913 | 0.827 |
|  | MLP | 0.833 | 0.795 | 0.872 | 0.669 | 0.925 | 0.827 |
|  | NB | 0.679 | 0.897 | 0.462 | 0.399 | 0.856 | 0.737 |
|  | PLS | 0.782 | 0.821 | 0.744 | 0.566 | 0.882 | 0.790 |
|  | RF | 0.859 | 0.744 | 0.974 | 0.738 | 0.906 | 0.841 |
|  | SVM | 0.859 | 0.769 | 0.949 | 0.730 | 0.905 | 0.845 |
|  | XGB | 0.872 | 0.846 | 0.897 | 0.745 | 0.916 | 0.868 |
| FP4C | ADA | 0.808 | 0.821 | 0.795 | 0.616 | 0.888 | 0.810 |
|  | DT | 0.782 | 0.692 | 0.872 | 0.573 | 0.853 | 0.761 |
|  | ET | 0.795 | 0.718 | 0.872 | 0.597 | 0.885 | 0.778 |
|  | KNN | 0.795 | 0.846 | 0.744 | 0.593 | 0.870 | 0.805 |
|  | LGBM | 0.808 | 0.821 | 0.795 | 0.616 | 0.846 | 0.810 |
|  | LR | 0.808 | 0.795 | 0.821 | 0.616 | 0.878 | 0.805 |
|  | MLP | 0.795 | 0.769 | 0.821 | 0.591 | 0.888 | 0.789 |
|  | NB | 0.808 | 0.641 | 0.974 | 0.653 | 0.878 | 0.769 |
|  | PLS | 0.808 | 0.846 | 0.769 | 0.617 | 0.882 | 0.815 |
|  | RF | 0.782 | 0.744 | 0.821 | 0.566 | 0.892 | 0.773 |
|  | SVM | 0.821 | 0.821 | 0.821 | 0.641 | 0.869 | 0.821 |
|  | XGB | 0.821 | 0.769 | 0.872 | 0.644 | 0.888 | 0.811 |
| Hybrid | ADA | 0.808 | 0.744 | 0.872 | 0.621 | 0.927 | 0.795 |
|  | DT | 0.808 | 0.769 | 0.846 | 0.617 | 0.804 | 0.800 |
|  | ET | 0.821 | 0.769 | 0.872 | 0.644 | 0.897 | 0.811 |
|  | KNN | 0.821 | 0.744 | 0.897 | 0.649 | 0.821 | 0.806 |
|  | LGBM | 0.833 | 0.769 | 0.897 | 0.672 | 0.921 | 0.822 |
|  | LR | 0.859 | 0.769 | 0.949 | 0.730 | 0.921 | 0.845 |
|  | MLP | 0.795 | 0.718 | 0.872 | 0.597 | 0.922 | 0.778 |
|  | NB | 0.859 | 0.769 | 0.949 | 0.730 | 0.856 | 0.845 |
|  | PLS | 0.833 | 0.744 | 0.923 | 0.678 | 0.903 | 0.817 |
|  | RF | 0.846 | 0.769 | 0.923 | 0.701 | 0.931 | 0.833 |
|  | SVM | 0.833 | 0.744 | 0.923 | 0.678 | 0.926 | 0.817 |
|  | XGB | 0.821 | 0.744 | 0.897 | 0.649 | 0.907 | 0.806 |
| KR | ADA | 0.808 | 0.718 | 0.897 | 0.626 | 0.915 | 0.789 |
|  | DT | 0.885 | 0.846 | 0.923 | 0.772 | 0.882 | 0.880 |
|  | ET | 0.846 | 0.769 | 0.923 | 0.701 | 0.895 | 0.833 |
|  | KNN | 0.821 | 0.769 | 0.872 | 0.644 | 0.821 | 0.811 |
|  | LGBM | 0.885 | 0.795 | 0.974 | 0.782 | 0.924 | 0.873 |
|  | LR | 0.859 | 0.795 | 0.923 | 0.724 | 0.919 | 0.849 |
|  | MLP | 0.846 | 0.795 | 0.897 | 0.696 | 0.922 | 0.838 |
|  | NB | 0.756 | 0.795 | 0.718 | 0.514 | 0.766 | 0.765 |
|  | PLS | 0.897 | 0.821 | 0.974 | 0.804 | 0.910 | 0.889 |
|  | RF | 0.872 | 0.795 | 0.949 | 0.753 | 0.921 | 0.861 |
|  | SVM | 0.859 | 0.769 | 0.949 | 0.730 | 0.918 | 0.845 |
|  | XGB | 0.859 | 0.744 | 0.974 | 0.738 | 0.931 | 0.841 |
| MACCS | ADA | 0.872 | 0.821 | 0.923 | 0.748 | 0.932 | 0.865 |
|  | DT | 0.846 | 0.795 | 0.897 | 0.696 | 0.865 | 0.838 |
|  | ET | 0.872 | 0.795 | 0.949 | 0.753 | 0.877 | 0.861 |
|  | KNN | 0.821 | 0.795 | 0.846 | 0.642 | 0.913 | 0.816 |
|  | LGBM | 0.846 | 0.795 | 0.897 | 0.696 | 0.928 | 0.838 |
|  | LR | 0.859 | 0.821 | 0.897 | 0.720 | 0.931 | 0.853 |
|  | MLP | 0.846 | 0.795 | 0.897 | 0.696 | 0.935 | 0.838 |
|  | NB | 0.718 | 0.821 | 0.615 | 0.445 | 0.847 | 0.744 |
|  | PLS | 0.821 | 0.692 | 0.949 | 0.663 | 0.874 | 0.794 |
|  | RF | 0.859 | 0.795 | 0.923 | 0.724 | 0.912 | 0.849 |
|  | SVM | 0.897 | 0.846 | 0.949 | 0.799 | 0.917 | 0.892 |
|  | XGB | 0.846 | 0.795 | 0.897 | 0.696 | 0.922 | 0.838 |
| PubChem | ADA | 0.833 | 0.769 | 0.897 | 0.672 | 0.891 | 0.822 |
|  | DT | 0.833 | 0.795 | 0.872 | 0.669 | 0.875 | 0.827 |
|  | ET | 0.846 | 0.795 | 0.897 | 0.696 | 0.937 | 0.838 |
|  | KNN | 0.872 | 0.846 | 0.897 | 0.745 | 0.872 | 0.868 |
|  | LGBM | 0.833 | 0.795 | 0.872 | 0.669 | 0.912 | 0.827 |
|  | LR | 0.872 | 0.846 | 0.897 | 0.745 | 0.931 | 0.868 |
|  | MLP | 0.897 | 0.821 | 0.974 | 0.804 | 0.936 | 0.889 |
|  | NB | 0.782 | 0.846 | 0.718 | 0.569 | 0.830 | 0.795 |
|  | PLS | 0.795 | 0.641 | 0.949 | 0.620 | 0.911 | 0.758 |
|  | RF | 0.846 | 0.769 | 0.923 | 0.701 | 0.946 | 0.833 |
|  | SVM | 0.885 | 0.821 | 0.949 | 0.776 | 0.907 | 0.877 |
|  | XGB | 0.833 | 0.821 | 0.846 | 0.667 | 0.914 | 0.831 |

## **Table S3** Average cross-validation performance of each ML methods over 12 different molecular descriptors.

| **Method** | **ACC** | **SN** | **SP** | **MCC** | **AUC** | **F1** |
| --- | --- | --- | --- | --- | --- | --- |
| ADA | 0.837 | 0.834 | 0.839 | 0.674 | 0.891 | 0.835 |
| DT | 0.847 | 0.832 | 0.862 | 0.695 | 0.881 | 0.844 |
| ET | 0.869 | 0.856 | 0.882 | 0.738 | 0.918 | 0.866 |
| KNN | 0.858 | 0.860 | 0.856 | 0.716 | 0.889 | 0.857 |
| LGBM | 0.860 | 0.862 | 0.859 | 0.721 | 0.924 | 0.860 |
| LR | 0.865 | 0.864 | 0.867 | 0.730 | 0.925 | 0.864 |
| MLP | 0.855 | 0.862 | 0.849 | 0.711 | 0.917 | 0.855 |
| NB | 0.773 | 0.756 | 0.790 | 0.557 | 0.824 | 0.765 |
| PLS | 0.807 | 0.786 | 0.827 | 0.617 | 0.890 | 0.801 |
| RF | 0.870 | 0.865 | 0.875 | 0.740 | 0.933 | 0.869 |
| SVM | 0.871 | 0.872 | 0.869 | 0.742 | 0.917 | 0.870 |
| XGB | 0.867 | 0.866 | 0.869 | 0.735 | 0.936 | 0.867 |

## **Table S4** Average independent test performance of each ML methods over 12 different molecular descriptors.

| **Method** | **ACC** | **SN** | **SP** | **MCC** | **AUC** | **F1** |
| --- | --- | --- | --- | --- | --- | --- |
| ADA | 0.819 | 0.763 | 0.874 | 0.643 | 0.893 | 0.808 |
| DT | 0.822 | 0.771 | 0.872 | 0.649 | 0.860 | 0.812 |
| ET | 0.825 | 0.761 | 0.889 | 0.656 | 0.902 | 0.813 |
| KNN | 0.826 | 0.786 | 0.865 | 0.657 | 0.868 | 0.819 |
| LGBM | 0.837 | 0.776 | 0.897 | 0.680 | 0.910 | 0.826 |
| LR | 0.832 | 0.782 | 0.883 | 0.669 | 0.902 | 0.824 |
| MLP | 0.829 | 0.780 | 0.878 | 0.663 | 0.914 | 0.820 |
| NB | 0.778 | 0.748 | 0.808 | 0.573 | 0.837 | 0.772 |
| PLS | 0.813 | 0.789 | 0.838 | 0.634 | 0.887 | 0.809 |
| RF | 0.844 | 0.769 | 0.919 | 0.698 | 0.920 | 0.832 |
| SVM | 0.846 | 0.784 | 0.908 | 0.699 | 0.906 | 0.836 |
| XGB | 0.840 | 0.772 | 0.908 | 0.688 | 0.915 | 0.828 |

## **Table S5** Average cross-validation performance of each molecular descriptor over 12 different ML methods.

| **Descriptor** | **ACC** | **SN** | **SP** | **MCC** | **AUC** | **F1** |
| --- | --- | --- | --- | --- | --- | --- |
| AP2D | 0.814 | 0.805 | 0.823 | 0.630 | 0.882 | 0.810 |
| Circle | 0.876 | 0.870 | 0.882 | 0.752 | 0.934 | 0.874 |
| CDK | 0.867 | 0.850 | 0.885 | 0.736 | 0.916 | 0.864 |
| CDKExt | 0.867 | 0.855 | 0.880 | 0.735 | 0.924 | 0.865 |
| CDKGraph | 0.831 | 0.796 | 0.865 | 0.666 | 0.891 | 0.822 |
| Estate | 0.800 | 0.805 | 0.794 | 0.600 | 0.863 | 0.799 |
| FP4 | 0.860 | 0.873 | 0.846 | 0.722 | 0.912 | 0.862 |
| FP4C | 0.829 | 0.827 | 0.830 | 0.659 | 0.883 | 0.826 |
| Hybrid | 0.888 | 0.876 | 0.900 | 0.777 | 0.926 | 0.886 |
| KR | 0.853 | 0.869 | 0.838 | 0.708 | 0.901 | 0.855 |
| MACCS | 0.849 | 0.859 | 0.838 | 0.700 | 0.904 | 0.850 |
| PubChem | 0.845 | 0.830 | 0.860 | 0.692 | 0.907 | 0.841 |

## **Table S6** Average independent test performance of each molecular descriptor over 12 different ML methods.

| **Descriptor** | **ACC** | **SN** | **SP** | **MCC** | **AUC** | **F1** |
| --- | --- | --- | --- | --- | --- | --- |
| AP2D | 0.824 | 0.752 | 0.895 | 0.661 | 0.872 | 0.809 |
| Circle | 0.847 | 0.776 | 0.919 | 0.703 | 0.922 | 0.836 |
| CDK | 0.850 | 0.782 | 0.919 | 0.709 | 0.913 | 0.839 |
| CDKExt | 0.842 | 0.791 | 0.893 | 0.690 | 0.913 | 0.834 |
| CDKGraph | 0.810 | 0.737 | 0.882 | 0.629 | 0.871 | 0.794 |
| Estate | 0.747 | 0.741 | 0.752 | 0.494 | 0.848 | 0.746 |
| FP4 | 0.824 | 0.795 | 0.853 | 0.657 | 0.900 | 0.821 |
| FP4C | 0.803 | 0.774 | 0.831 | 0.610 | 0.876 | 0.796 |
| Hybrid | 0.828 | 0.754 | 0.902 | 0.664 | 0.895 | 0.815 |
| KR | 0.849 | 0.784 | 0.914 | 0.707 | 0.894 | 0.840 |
| MACCS | 0.842 | 0.797 | 0.887 | 0.690 | 0.904 | 0.836 |
| PubChem | 0.844 | 0.797 | 0.891 | 0.694 | 0.905 | 0.836 |

## **Table S7** Cross-validation and independent test results of MetaCGRP over 10 different training and independent test datasets

| **Experiment** | **Cross-validation** | | | | **Independent test** | | | |
| --- | --- | --- | --- | --- | --- | --- | --- | --- |
|  | **ACC** | **MCC** | **AUC** | **F1** | **ACC** | **MCC** | **AUC** | **F1** |
| 1 | 0.928 | 0.856 | 0.953 | 0.925 | 0.897 | 0.798 | 0.955 | 0.905 |
| 2 | 0.928 | 0.857 | 0.961 | 0.928 | 0.897 | 0.800 | 0.946 | 0.897 |
| 3 | 0.922 | 0.843 | 0.939 | 0.921 | 0.897 | 0.799 | 0.958 | 0.895 |
| 4 | 0.925 | 0.850 | 0.937 | 0.925 | 0.897 | 0.799 | 0.955 | 0.895 |
| 5 | 0.912 | 0.824 | 0.941 | 0.913 | 0.897 | 0.796 | 0.957 | 0.882 |
| 6 | 0.935 | 0.870 | 0.961 | 0.935 | 0.885 | 0.771 | 0.925 | 0.877 |
| 7 | 0.918 | 0.837 | 0.943 | 0.920 | 0.910 | 0.820 | 0.956 | 0.896 |
| 8 | 0.935 | 0.870 | 0.962 | 0.935 | 0.885 | 0.771 | 0.927 | 0.877 |
| 9 | 0.928 | 0.856 | 0.964 | 0.929 | 0.873 | 0.749 | 0.916 | 0.861 |
| 10 | 0.915 | 0.830 | 0.961 | 0.915 | 0.885 | 0.772 | 0.931 | 0.880 |
| Average | 0.925 | 0.849 | 0.952 | 0.925 | 0.892 | 0.788 | 0.943 | 0.886 |
| STD | 0.008 | 0.016 | 0.011 | 0.008 | 0.010 | 0.021 | 0.016 | 0.013 |

## **Table S8** List of 19 important features of PF_FS along with their SHAP values

| **#** | **Feature** | **MeanSHAP** |
| --- | --- | --- |
| 1 | XGB-Hybrid | 0.0820 |
| 2 | XGB-PubChem | 0.0430 |
| 3 | RF-Circle | 0.0392 |
| 4 | MLP-CKDExt | 0.0247 |
| 5 | DT-PubChem | 0.0190 |
| 6 | PLS-Estate | 0.0141 |
| 7 | SVM-KR | 0.0083 |
| 8 | RF-Estate | 0.0026 |
| 9 | LR-PubChem | 0.0009 |
| 10 | PLS-Circle | 0.0008 |
| 11 | KNN-Estate | 0.0007 |
| 12 | ADA-FP4 | 0.0007 |
| 13 | LR-CKDGraph | 0.0007 |
| 14 | XGB-MACCS | 0.0006 |
| 15 | DT-Hybrid | 0.0006 |
| 16 | LGBM-CKD | 0.0005 |
| 17 | MLP-PubChem | 0.0005 |
| 18 | SVM-Hybrid | 0.0005 |
| 19 | DT-Circle | 0.0005 |

## **Table S9** List of top-20 important Pubchem features along with their SHAP values

| **#** | **Feature** | **MeanSHAP** |
| --- | --- | --- |
| 1 | Pubchem675 | 0.1955 |
| 2 | Pubchem602 | 0.1680 |
| 3 | Pubchem14 | 0.1447 |
| 4 | Pubchem375 | 0.1209 |
| 5 | Pubchem443 | 0.1104 |
| 6 | Pubchem759 | 0.1099 |
| 7 | Pubchem350 | 0.0854 |
| 8 | Pubchem697 | 0.0783 |
| 9 | Pubchem578 | 0.0766 |
| 10 | Pubchem690 | 0.0763 |
| 11 | Pubchem618 | 0.0742 |
| 12 | Pubchem537 | 0.0682 |
| 13 | Pubchem494 | 0.0678 |
| 14 | Pubchem735 | 0.0668 |
| 15 | Pubchem713 | 0.0595 |
| 16 | Pubchem398 | 0.0515 |
| 17 | Pubchem492 | 0.0481 |
| 18 | Pubchem365 | 0.0473 |
| 19 | Pubchem259 | 0.0396 |
| 20 | Pubchem336 | 0.0377 |

## **Table S10** Physicochemical properties of the top-five compounds and the FDA-approved drugs*

| **Compound** | **Compound Name** | **MW** | **AlogP** | **HBDon** | **HBAc** | | **TPSA** | **nRotB** |
| --- | --- | --- | --- | --- | --- | --- | --- | --- |
| Top-five | Clerosterol 3-glucoside | 574.8 | 6.14 | 4 | 6 | 99.4 | | 9 |
|  | Stigmasterol 3-glucoside | 574.8 | 5.89 | 6 | 4 | 99.38 | | 8 |
|  | Sennoside D | 848.7 | -3.86 | 19 | 12 | 330.89 | | 9 |
|  | Sennoside C | 848.7 | -3.86 | 19 | 12 | 330.89 | | 9 |
|  | Stantalin A | 582.5 | -0.18 | 10 | 5 | 155.14 | | 6 |
| FDA-approved | Ubrogepant | 549.5 | 2.05 | 8 | 2 | 103.23 | | 6 |
|  | Rimegepant | 534.5 | 0.95 | 9 | 2 | 112.62 | | 5 |
|  | Zavegepant | 638.8 | 0.76 | 11 | 3 | 112.62 | | 9 |
|  | Atogepant | 603.5 | 1.77 | 8 | 2 | 103.23 | | 6 |

*Data derived from CDK package in Rstudio
